# High-throughput screening identified selective inhibitors of exosome biogenesis and secretion: A drug repurposing strategy for advanced cancer

**DOI:** 10.1038/s41598-018-26411-7

**Published:** 2018-05-25

**Authors:** Amrita Datta, Hogyoung Kim, Lauren McGee, Adedoyin E. Johnson, Sudha Talwar, Juan Marugan, Noel Southall, Xin Hu, Madhu Lal, Debasis Mondal, Marc Ferrer, Asim B. Abdel-Mageed

**Affiliations:** 1 Departments of Urology, Tulane University School of Medicine, New Orleans, LA 70112 United States; 2 Departments of Pharmacology, Tulane University School of Medicine, New Orleans, LA 70112 United States; 3 Tulane Cancer Center, Tulane University School of Medicine, New Orleans, LA 70112 United States; 4 Division of Preclinical Innovation, National Center for Advancing Translational Sciences (NCATS), National Institutes of Health, Bethesda, Maryland 20850 United States

## Abstract

Targeting exosome biogenesis and release may have potential clinical implications for cancer therapy. Herein, we have optimized a quantitative high throughput screen (qHTS) assay to identify compounds that modulate exosome biogenesis and/or release by aggressive prostate cancer (PCa) CD63-GFP-expressing C4-2B cells. A total of 4,580 compounds were screened from the LOPAC library (a collection of 1,280 pharmacologically active compounds) and the NPC library (NCGC collection of 3,300 compounds approved for clinical use). Twenty-two compounds were found to be either potent activators or inhibitors of intracellular GFP signal in the CD63-GFP-expressing C4-2B cells. The activity of lead compounds in modulating the secretion of exosomes was validated by a tunable resistive pulse sensing (TRPS) system (qNano-IZON) and flow cytometry. The mechanism of action of the lead compounds in modulating exosome biogenesis and/or secretion were delineated by immunoblot analysis of protein markers of the endosomal sorting complex required for transport (ESCRT)-dependent and ESCRT-independent pathways. The lead compounds tipifarnib, neticonazole, climbazole, ketoconazole, and triademenol were validated as potent inhibitors and sitafloxacin, forskolin, SB218795, fenoterol, nitrefazole and pentetrazol as activators of exosome biogenesis and/or secretion in PC cells. Our findings implicate the potential utility of drug-repurposing as novel adjunct therapeutic strategies in advanced cancer.

## Introduction

Exosomes are nanovesicles (30–150 nm) that are generated by inward budding of the multivesicular bodies (MVBs) and secreted extracellularly upon fusion of the MVBs with the plasma membrane^1^. Exosomes are detected in a variety of biological fluids, including blood, urine, saliva, amniotic fluid, malignant ascites and breast milk, etc.^2^. Because they are important mediators of intercellular communication in many physiological and pathological processes, exosomes have been implicated in a broad range of functional activities, such as protein clearance, immunity, infections, signaling, and cancer. The exosomal ‘cargo’ content (lipids, metabolites, proteins, and nucleic acids, including non-coding RNAs) can modify the physiological state of recipient cells. In particular, the tumor-derived exosomes are implicated in promoting tumor progression, angiogenic switch, and immune escape by paracrine subversion of local and distant microenvironments^3^. Their roles in various stages of metastasis, including the induction of migration, invasion and pre-metastatic niche formation, have been well-documented in various human neoplastic diseases, including pancreatic cancer, melanoma, breast cancers^4–7^. We have recently documented a role for prostate-cancer-derived exosomes in the oncogenic reprogramming of patients’ procured tumor-tropic adipose stem cells^8^. Indeed, cell surface proteins and exosome selective biomarkers (CD9, CD81, CD63, etc.) can be instrumental in identifying and sorting of cell-type specific exosomes, and these markers may be used in the targeting and uptake of exosomes by the recipient cells. Furthermore, exosome-specific markers may be effectively used to screen for potent lead compounds that modulate exosome biogenesis, release and/or uptake.

It is well-known that exosomes are secreted by tumor cells, and their circulating levels are elevated in cancer patients with aggressive disease^9^. To this end, several recent strategies have been established towards exploiting tumor-derived exosomes as liquid biomarkers for cancer diagnosis and prognosis^10–14^. However, despite their unequivocal roles in disease progression, little progress has been made in targeting exosome biogenesis, release and/or uptake as novel adjuvants for cancer therapy. An affinity plasmapheresis platform to decrease systemic levels of HER2-positive exosomes and inhibit the progression of HER2-positive breast tumors has been reported as a possible adjunct therapeutic approach^15^. This study provided promising evidence that the removal of tumor-derived exosomes from the circulatory system could be a novel direction in the treatment of cancer. However, there has been no significant progress in the identification of pharmacological agents that potently inhibit exosome biogenesis, secretion and/or uptake by the tumor cells and/or the tumor-associated stroma. Thus, a quantitative high throughput screen (qHTS) to identify lead therapeutic agents that suppress circulating tumor-derived exosome levels would be of paramount significance in preventing both disease progression and poor prognostic outcomes^16^.

Drug repositioning is becoming an attractive approach to rapidly identify novel targets for cancer therapy^17^. The translational value of potent modulators of exosome biogenesis, secretion and/or uptake can be realized by qHTS assays of existing drug libraries. We hypothesized that the NIH Chemical Genomics Center (NCGC) pharmaceutical collection (collectively known as NPC) is a comprehensive resource of 3,300 approved and registered molecular entities (MEs) enabling repurposing and chemical genomics^18^ and the Sigma Life Science’s Library of Pharmacologically Active Compounds (LOPAC^1280^), is a biologically annotated collection of 1,280 compounds, including inhibitors, receptor ligands, pharma-developed tools, and approved drugs^19^. Towards this goal, we generated a C4-2B cell-based CD63-GFP imaging assay to implement a dose-response qHTS of these two library collections. Selected lead compounds from the screen were further validated for their ability to modulate exosome biogenesis and secretion using qNano IZON and MACSQuant. The mechanism of action of leads was delineated by biological assays in aggressive cancer cells.

## Results

### Development and optimization of the qHTS-compatible exosome biogenesis assay

The pCMV-CD63-GFP expression plasmid was used to generate a stable C4-2B cell line expressing the exosomal marker CD63 fused with GFP. C4-2B-CD63-GFP cells seeded in a 1536-well plate were used to optimize the GFP signal (including optimization of cell seeding densities, media conditions, incubation times, and plate coating) for assay development for qHTS studies (Supplementary Fig. S1). In Supplementary Fig. S1A, GFP fluorescence was visible in the poly-D-lysine coated plate as compared with the non-coated plate. Fluorescence microscopy showed that the expression of CD63-GFP co-localized with the CD63 immunostaining (Supplementary Fig. S1B). Optimized assay conditions included the coating of the plates with different coating solutions, seeding 2,000 cells/well, using low serum media, and treatment of the cells with Manumycin A (MA), a farnesyl transferase inhibitor for 96 hrs (Supplementary Fig. S1C). The effect of MA, a previously reported inhibitor of exosome biogenesis and secretion was used as a control (REF). An additional compound GW4869 (a neutral sphingomyelinase inhibitor) known to interfere with exosome secretion and vesicle trafficking, in general, was also used to validate the assay (Supplementary Fig. S1D). The time-line for the biogenesis assay is shown in Fig. S2A. And these optimization strategies produced assay plates with Z′-factors of ~0.6 (Supplementary Fig. S2B) indicating that the assay was robust for 1536-well qHTS of exosome biogenesis inhibitors.

### qHTS for biogenesis modulators using the CD63-GFP expressing C4-2B cells

**Figure 1:**
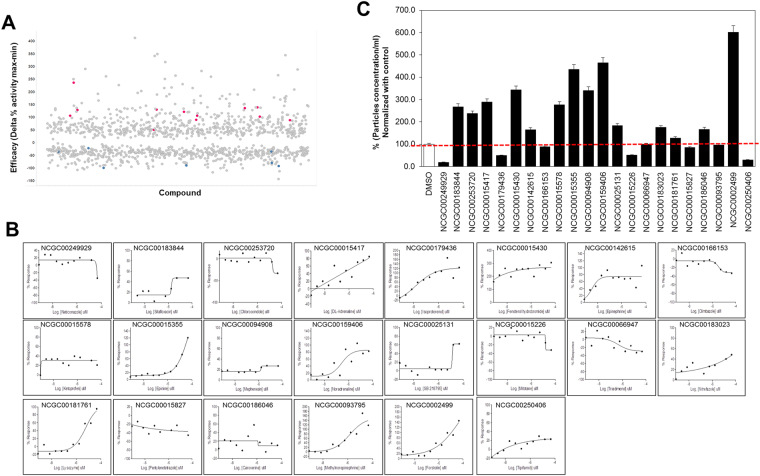
Validation of inhibitors and activators of exosome biogenesis using the qHTS and the quantitative qNano IZON particle analysis: (**A**) Scatter plot of the % efficacy (the difference in maximum and minimum activity in the dose-response) of all the compounds with activity in the qHTS (CRC ±1, ±2, ±3). Highlighted in red are compounds validated to increase CD63-GFP signal and, in blue, those validated to decrease CD63-GFP. (**B**) CRC curves for 22 compounds of the 22 hits obtained from the qHTS assay are shown here. (**C**) C4-2B-CD63-GFP cells were maintained overnight in exosome free RPMI media and then treated with the qHTS hits (compounds) or DMSO (vehicle control) for 48 hours. Treatments were at concentrations of 1 μM for neticonazole and tipifarnib and 10 μM for all other compounds. The exosomes in the conditioned media were isolated, filtered thorough 0.22 μM filter and analyzed by the qNano IZON system using NP-100 nanopore. A detailed description of the protocol is described in “Materials and Methods” section. qNano IZON particle quantitative analysis (NP-100 nanopore) depicting a significant decrease in exosome concentrations (50–200 nm size) in the CM of C4-2B-CD63-GFP cells treated with validated compounds compared to vehicle-treated controls.

Two libraries were screened to investigate the modulatory effects of a variety of compounds on exosome biogenesis: the LOPAC, a commercially available collection of 1280 pharmacologically active compounds and the NPC (NCATS Pharmaceutical Collection), a collection of 2800 clinically approved compounds. The screen used CD63-GFP as a readout, as shown in Fig. 1A; highlighted in red are compounds validated to increase CD63-GFP signal and, in blue, those validated to decrease CD63-GFP. Additionally, we performed a dose-response experiment using the compounds at 46 μM and 5-fold dilutions (Supplementary Fig. S2C). The assays also included a viability counter screen, a Cell Titer Glo® reagent that measures adenosine triphosphate (ATP) as readout for cell proliferation, to determine whether any decreases in GFP signal are primarily due to a decrease in CD63-GFP biogenesis and not due to a reduction in cell viability. Analyses of the results indicated that the CD63-GFP assay had enough dynamic range to detect both inhibitors and activators of CD63-GFP exosome biogenesis. Compounds were selected based on the curve response class (CRC) algorithm for a dose response screening, which enables the selection of compounds based on both potency (EC_50_) and efficacy (difference between the maximum and minimum percent of activity and the dose-response of each compound tested). Selected compounds had robust dose responses curves determined by CRC values of robust CRCs 1.1, 2.1, 1.2, 2.2 for activators, and −1.1, −2.1, −1.2, −2.2 for inhibitors, and had no activity in the viability assays (CRC 4). Supplementary Figure S2D depicts schematic examples of the curves with different CRC scores. To further confirm activity and to generate more accurate IC_50_ values, 128 exosome biogenesis activators and inhibitors were selected and re-tested in the same assay using 11-point dose responses. We selected activators with the notion that the blocking of exosome secretion would increase their levels inside the cells, resulting in increases in the GFP signal. The 22 confirmed compounds (Table 1) produced a robust and reproducible CRC score on CD63-GFP signal (Fig. 1B) and, thus, were next subjected to further validation to determining whether they affected exosome biogenesis or secretion.

**Table 1:** qHTS hits selected for validation.

NCATS No.	NCGC No.	Compound name	CRC values	Efficacy (Delta % activity max-min)
1	NCGC00249929-01	Neticonazole	1.2	45
2	NCGC00183844-04	Sitafloxacin	−2.2	−38
3	NCGC00253720-01	Chloroxindole	1.2	90
4	NCGC00015417-04	Racepinephrine	1.1	126
5	NCGC00179436-03	Isoproterenol	1.1	143
6	NCGC00015430-06	Fenoterol Hydrobromide	2.1	238
7	NCGC00142615-05	L-epinephrine bitartate	1.1	91
8	NCGC00166153-03	Climbazole	1.1	135
9	NCGC00015578-14	Ketoprofen	−2.2	−44
10	NCGC00015355-04	N-Methyldopamine	2.1	106
11	NCGC00094908-05	Mephensin	−2.2	−94
12	NCGC00159406-12	Norepinephrine Bitartrate	1.1	108
13	NCGC00025131-04	SB 218795	2.2	55
14	NCGC00015226-13	Mitotane	−1.4	−33
15	NCGC00066947-05	Triadimenol	2.2	59
16	NCGC00183023-01	Nitrefazole	−2.2	−76
17	NCGC00181761-01	Lysozyme	2.1	101
18	NCGC00015827-11	Pentylenetetrazole	−2.1	−97
19	NCGC00186046-01	Caroverine	−2.1	−91
20	NCGC00093795-05	Methylnorepinephrine	1.1	127
21	NCGC00024996-31	Forskolin	1.1	248
22	NCGC00250406-04	Tipifarnib	1.2	49

Twenty-two selected hits are tabulated along with the National Institutes of Health (NIH) Chemical Genomics Center (NCGC) number, compound name, CRC values and maximal response values.

### Validation of qHTS leads

Based on our recent study^20^, we employed a modified workflow for the isolation of exosomes (Supplementary Fig. S3) and their detection using the qNano IZON system (NP100 nanopore) to validate the ability of the 22 qHTS assay hits to modulate the particle concentration, the mean diameter (nm) and diameter mode (nm) secreted by the C4-2B-CD63-GFP cells (Supplementary Table S1). The two most potent inhibitors from the qHTS assay, neticonazole, and tipifarnib were used at a concentration of 1 μM while the rest of the lead compounds were used at a concentration of 10 μM. Figure 1C shows the effects of the 22 lead compounds on the exosome concentration using qNano IZON system. Data are represented as a percentage of exosomal particles in cell supernatants after normalization to the vehicle-treated controls. Neticonazole (19%), tipifarnib (30%), isoproterenol (49.4%), mitotane (50.7%), pentetrazol (85%) and climbazole (88%) were found to be potent inhibitors of exosome secretion. Also, we selected compounds that showed increased GFP signal in our qHTS assays as activators of exosome secretion. Sitafloxacin, chloroxindole, D,L-adrenaline, fenoterol, epinephrine, ketoprofen, methyldopamine, mephenesin, norepinephrine, SB2187595, nitrefazole, caroverine, forskolin increased exosome concentrations. In line with our qHTS data, the lead compounds had similar inductive or suppressive effects on exosome secretion from the C4-2B cells. However, additional biological studies are required to further validate the activators of biogenesis/secretion exosomes.

**Figure 2:**
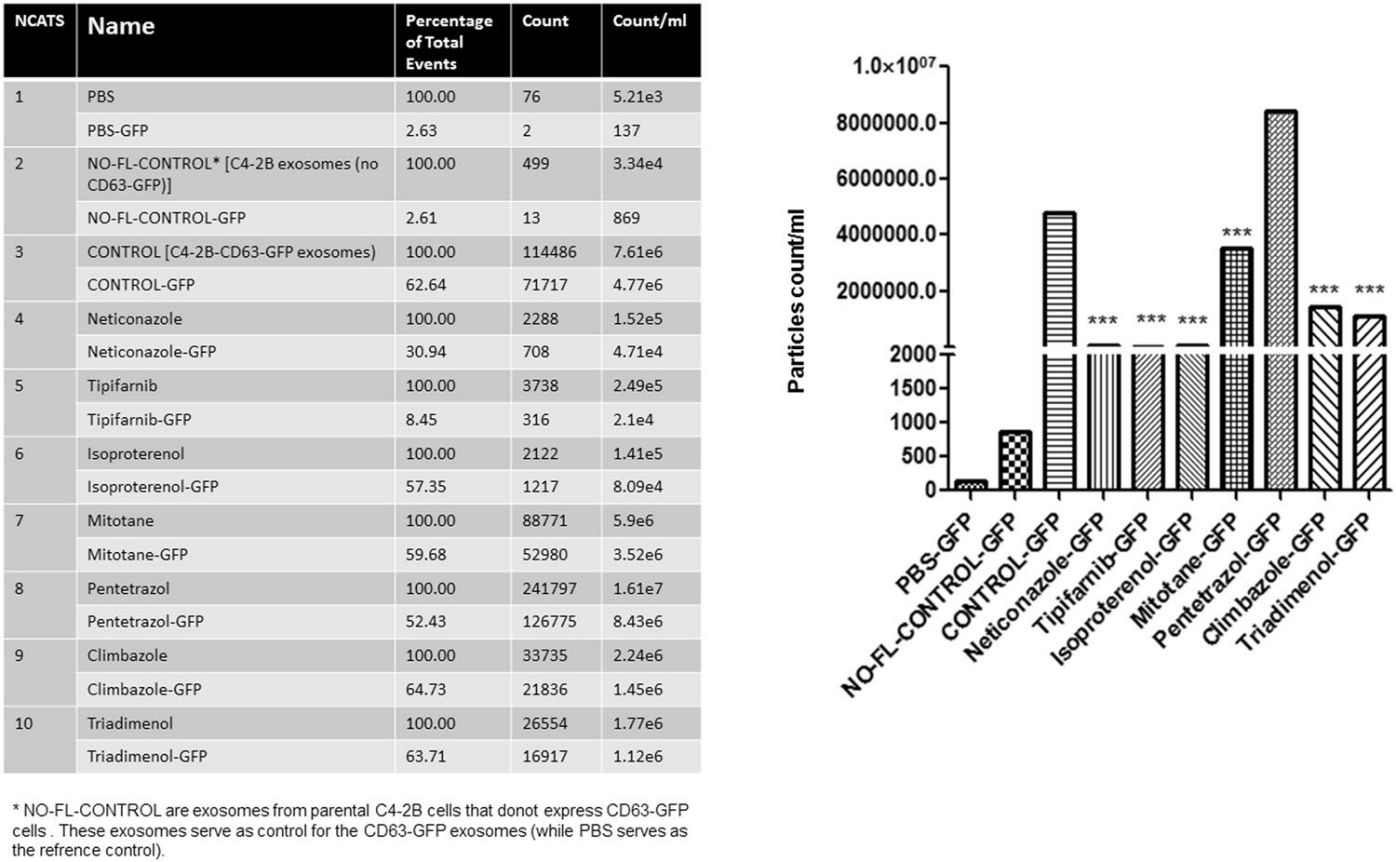
Validation of inhibitors of exosome biogenesis using the MACSQuant Analyzer 10 Flow Cytometer: The exosomes in the conditioned media were isolated and analyzed by the MACSQuant Analyzer 10 Flow Cytometer as described in the “Materials and Methods” section. Treatments were at concentrations of 1 μM for neticonazole and tipifarnib and 10 μM for all other compounds. All the compounds identified as inhibitors using the qNano IZON were validated as potent inhibitors of exosome biogenesis by the MACSQuant, as well (except pentetrazol). The data is represented as the percentage of exosomes and has been normalized to the control sample (100%). Neticonazole (19%), tipifarnib (30%), isoproterenol (49.4%), mitotane (50.7%), pentetrazol (85%) and climbazole (88%) are potent inhibitors of exosome biogenesis. Three controls were used in this experiment. PBS without EVs was used as a reference control. The NO-FL-CONTROL are exosomes from parental C4-2B cells that did not express CD63-GFP cells. Exosomes from DMSO treated C4-2B-CD63-GFP is used as GFP control and PBS served as a reference control. Three samples determined to be activators of exosome biogenesis by the qNano-IZON were also tested by the MACSQuant Analyzer validated to be activators (Fig. S5). ***Denotes significance at p < 0.05 compared to controls and was calculated using GraphPad Prism.

The qNano IZON results were further corroborated by the MACSQuant Analyzer and/or NanoFACS (Bio-Rad). Exosomes from C4-2B-CD63-GFP treated with DMSO and exosomes released from the parental C4-2B cells were used as controls. The percentage of total events, count and count/mL are shown in Fig. 2 (left panel). With exception of pentetrazol and nitrefazole, Fig. 2 (right panel) shows that neticonazole, tipifarnib, isoproterenol, climbazole and triademenol identified as inhibitors by the qNano IZON system were validated as potent inhibitors of exosome biogenesis by the MACSQuant analyzer. Additionally, sitafloxacin, forskolin and ketoprofen, pentetrazol and nitrefazole determined to be activators of exosome biogenesis by the qNano IZON were also validated by BioRad’s NanoFACS (Supplementary Fig. S4). Cell viability studies were carried out to determine if the lead compounds show any cytotoxicity at the concentrations used to modulate exosome biogenesis and/or secretion. Except for tipifarnib, all of the other lead drugs showed >80% cell survival following exposure to 10 μM dose for 72 hours (Supplementary Fig. S5A). In all experiments, tipifarnib was used at ≤250 nM, which had no effect on cell viability (Supplementary Fig. S5B).

### Tipifarnib inhibited exosome biogenesis by PCa cells

**Figure 3:**
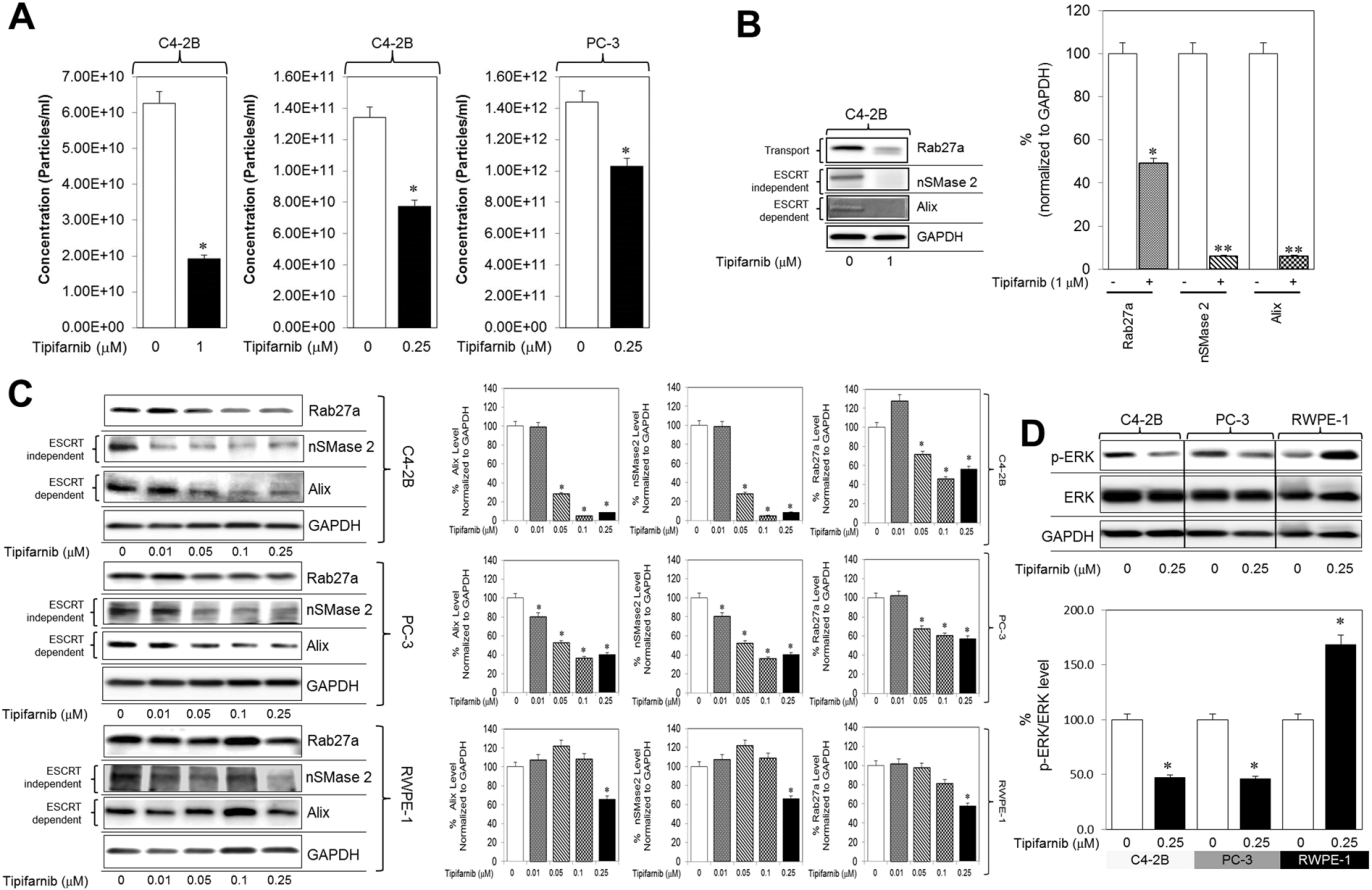
Tipifarnib inhibits exosome biogenesis and secretion via both ESCRT dependent and independent pathways and disrupts RAS signaling. (**A**) C4-2B and PC-3 cells were maintained overnight in exosome free DMEM media and then treated with tipifarnib or DMSO (vehicle control) for 48 hours. The concentration of exosomes was measured by the qNano IZON. Tipifarnib shows a significant decrease in the concentration of exosomes in C4-2B cells both at 0.25 and 1 μM as well as in the PC-3 cells at 0.25 μM. (**B**) Tipifarnib at a 1 μM concentration significantly inhibited the protein concentration of Alix, nSMase2, and Rab27a in C4-2B cells. Full blots are presented in Supplementary Fig. S6. (**C**) Tipifarnib significantly inhibited the protein concentration of Alix, nSMase2, and Rab27a in a dose-dependent manner in C4-2B and PC-3 cells but not in the normal RWPE-1 cells. Full blots are presented in Supplementary Fig. S6. (**D**) Tipifarnib significantly inhibited the activation of p-ERK (downstream effector molecule of the Ras/Raf/ERK signaling pathway) but not total ERK in C4-2B and PC-3 cells but not in the normal RWPE-1 cells. Full blots are presented in Supplementary Fig. S6. Mean values and standard errors were derived from four independent experiments. *Denotes significance at p < 0.05 compared to controls and was calculated using GraphPad Prism.

Tipifarnib was found to be one of the most potent exosome inhibitors in qHTS assays. Due to its potent farnesyl transferase inhibitor (FTI) effect, tipifarnib shows inhibition of cell growth or angiogenesis, and induction of apoptosis^21–24^. The exosome inhibitory effects of tipifarnib were evident even in the nanomolar range, which was then used in subsequent validation and molecular mechanism studies. The qNano IZON analysis of exosomes released from PCa cells were carried out following exposure to 0.25–1 μM of tipifarnib. A significant decrease in total particle concentrations was observed in the supernatants of both C4-2B and PC-3 cells (Fig. 3A).

The endosomal sorting complex required for transport (ESCRT) machinery is pivotal to exosome biogenesis, cargo sorting and secretion^25^. Other ESCRT-independent pathways, i.e., lipid-mediated and tetraspanin-mediated, also play major roles^25,26^. To unravel the underpinning mechanisms of tipifarnib, we examined the protein expression involved in both ESCRT-dependent (Alix) and ESCRT-independent (nSMase2) exosome biogenesis and transport (Rab27a)^26^. A significant decrease in Alix, nSMase2, and Rab27a was observed in C4-2B cells treated with tipifarnib (Fig. 3B). Subsequent experiments were carried out to compare the effects of treatment with tipifarnib for 48 hours in the RWPE-1, C4-2B, and PC-3 cells. We showed that tipifarnib (0–250 nM) causes a dose-dependent decrease in Alix, nSMase2, and Rab27a in both C4-2B and PC-3 cells, but not in the RWPE-1 cells (Fig. 3C). Thus, the exosome inhibitory effects of tipifarnib may occur via multiple pathways and may be selective for cancer cells.

Activation of Ras oncogene has been reported in PCa^27^. Ras farnesylation is required for the activation and phosphorylation of downstream ERK (p-ERK), but FTI decreases Ras activation and ERK phosphorylation^28^. We recently showed that both ERK inhibitor (U0126) and the FTI, Manumycin A (MA) decrease exosome biogenesis in PCa cells^20^. Therefore, we investigated whether tipifarnib, an FTI, can similarly decrease Ras-mediated ERK activation in RWPE-1, C4-2B, and PC-3 cells. Immunoblot analysis demonstrated a significant decrease in pERK levels following exposure to increasing concentrations of tipifarnib, in both C4-2B and PC-3 cells, but not in RWPE-1 cells (Fig. 3D). This established a link between tipifarnib inhibition of exosome biogenesis (and secretion) and disruption of Ras/Raf/ERK signaling pathways and the suppression of both ESCRT-dependent, ESCRT-independent pathways in PCa cells.

### Azoles modulate exosome biogenesis in PC cells

**Figure 4:**
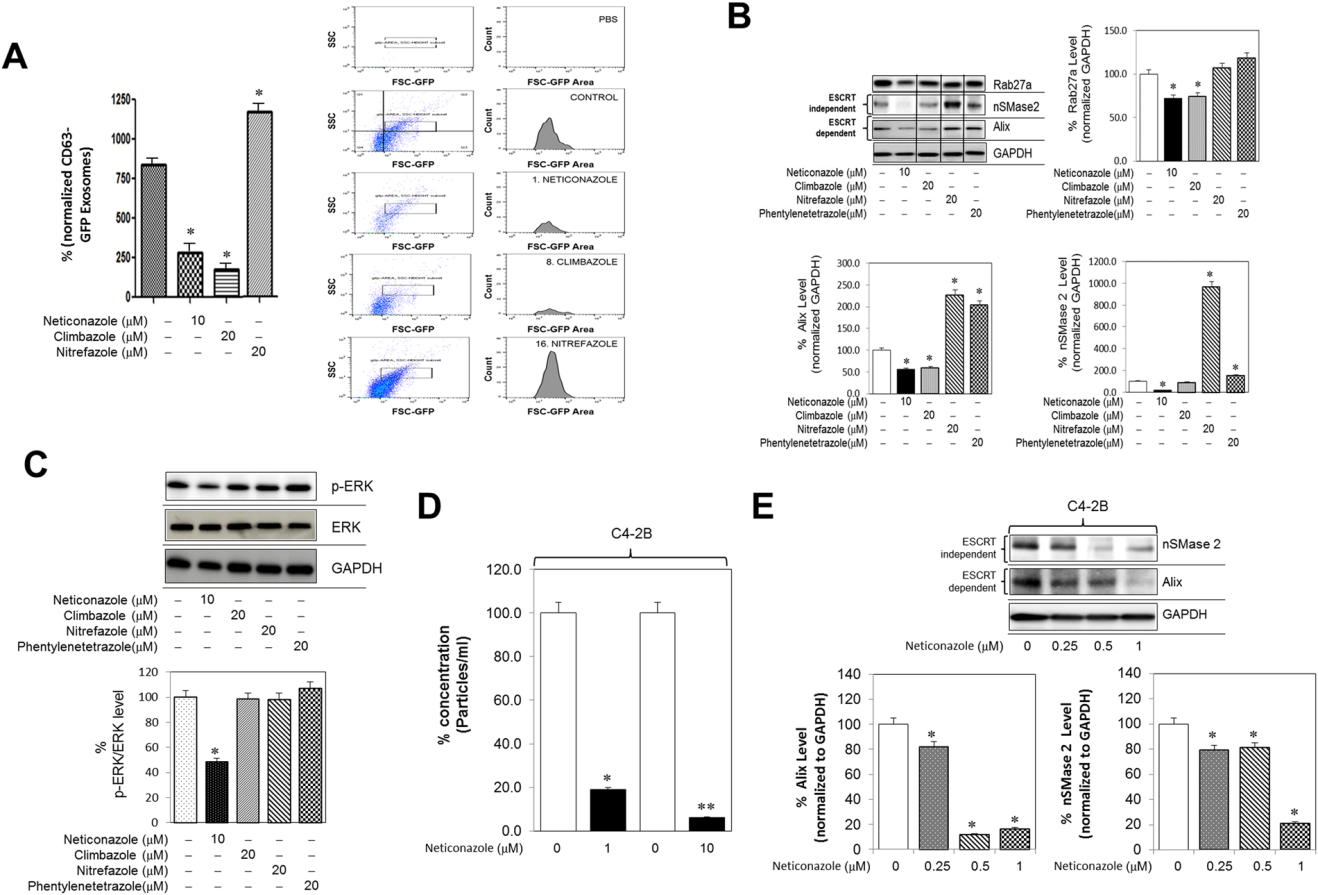
Azoles modulate exosome biogenesis through ESCRT dependent and independent pathway. (**A**) C4-2B-CD63-GFP were maintained overnight in exosome free DMEM media and then treated with the azoles or DMSO (vehicle control) for 48 hours. The concentration of exosomes was measured by the NanoFACS system as described in the “Materials and Methods” section. The concentration of exosomes from neticonazole and climbazole -treated cells was significantly lower than DMSO (vehicle) while the concentration of exosomes from nitrefazole treated cells was significantly higher. PBS without EVs was used as a reference control. (**B**) Neticonazole significantly inhibited the protein concentration of Alix, nSMase2 and Rab27a in C4-2B cells and climbazole significantly inhibited the protein concentration of Alix, and Rab27a but not nSMase2. Nitrefazole and pentetrazol showed a significant increase in Alix and nSMase2, but not in Rab27a. Full blots are presented in Supplementary Fig. S6. (**C**) Neticonazole significantly inhibited the activation of p-ERK (downstream effector molecule of the Ras/Raf/ERK signaling pathway) but not total ERK in C4-2B. Full blots are presented in Supplementary Fig. S6. (**D**) Neticonazole significantly inhibited the concentration of exosomes measured by qNano IZON in a dose-dependent manner in C4-2B cells. (**E**) Neticonazole significantly inhibited the protein concentration of Alix and nSMase2 in a dose-dependent manner in C4-2B cells. Full blots are presented in Supplementary Fig. S6. Mean values and standard errors were derived from four independent experiments. *Denotes significance at p < 0.05 compared to controls and was calculated using GraphPad Prism.

Our qHTS assays uncovered a number of azole compounds with potent effects in modulating exosome production from PCa cells (Figs 1 and 2; Table 1. Importantly, a number of these azoles have been clinically approved for other indications. For example, the lead imidazole compounds: ketoconazole, neticonazole, and climbazole are potent antifungal agents^29^. The modified imidazole (nitroimidazoles), nitrefazole is an approved aldehyde dehydrogenase (ALDH) inhibitor that is used to treat alcoholism^30^ and the lead tetrazole compound, phenylenetetrazole (pentetrazol) has been used as a circulatory and respiratory stimulant^31^. We first carried out validation experiments using both qNano IZON (Fig. 1C) and MACSQuant (Fig. 2), followed by molecular studies to delineate their mechanisms of action on exosome biogenesis/secretion. The C4-2B-CD63-GFP cells were treated with individual azole compounds for 48 hours and the exosomes isolated from the CM was analyzed using a MACSQuant (Fig. 4A). The qNano IZON assays showed that exposure to neticonazole significantly decreased and to nitrefazole (20 µM) significantly increased exosome concentration released by the C4-2B cells (Fig. 1C). In studies using the MACSQuant, we compared the efficacies of neticonazole, climbazole, and nitrefazole (Fig. 4A). Similar to the qNano IZON data, MACSQuant analysis revealed that neticonazole significantly decreased and nitrefazole significantly increased the concentration of GFP-labeled exosomes released by the C4-2B cells (Fig. 4A). In contrast to the qNano IZON analysis, the MACSQuant analysis clearly showed that climbazole (20 µM) significantly decreases exosome secretion (Fig. 4A).

To delineate the molecular mechanisms by which the lead azole compounds modulate exosome biogenesis/secretion, we measured Alix, nSMase2 and Rab27a protein levels in C4-2B following treatment with the Azoles. While both neticonazole and climbazole decreased the levels of both Alix and Rab27a, neticonazole significantly decreased nSMase2 levels as well (Fig. 4B). Neither pentylenetetrazol nor nitrefazole altered the levels of Rab27a; rather they caused an increase in Alix and nSMase2 (Fig. 4B). These findings corroborated exosome-induced production by nitrefazole C4-2B cells. Moreover, while neticonazole caused a significant inhibition in p-ERK levels, both nitrefazole and pentetrazol significantly increased p-ERK, which was consistent with their effects on Alix and nSMase2 (Fig. 4C). These findings implicated that the azole compounds may function via different mechanisms to modulate the biogenesis and release of exosomes by the C4-2B cells.

Due to the consistent data obtained with neticonazole, we evaluated its concentration dependent effects in inhibiting exosome production by measuring both particle concentration, and Alix and nSMase2 levels in C4-2B cells (Fig. 4E). The qNano IZON analysis revealed that neticonazole exhibits a potent and dose-dependent inhibition of exosome release from C4-2B cells (Fig. 4D). In addition, neticonazole, at sub-micromolar concentrations (0.25–1 μM), effectively suppressed both nSMase2 and Alix expression in these cells. These findings established the potent exosome-inhibitory effects of neticonazole not only via ESCRT-independent and ESCRT-dependent pathways but also by targeting Ras/ERK signaling.

### The clinically approved Ketoconazole potently suppressed exosome production by PCa cells

**Figure 5:**
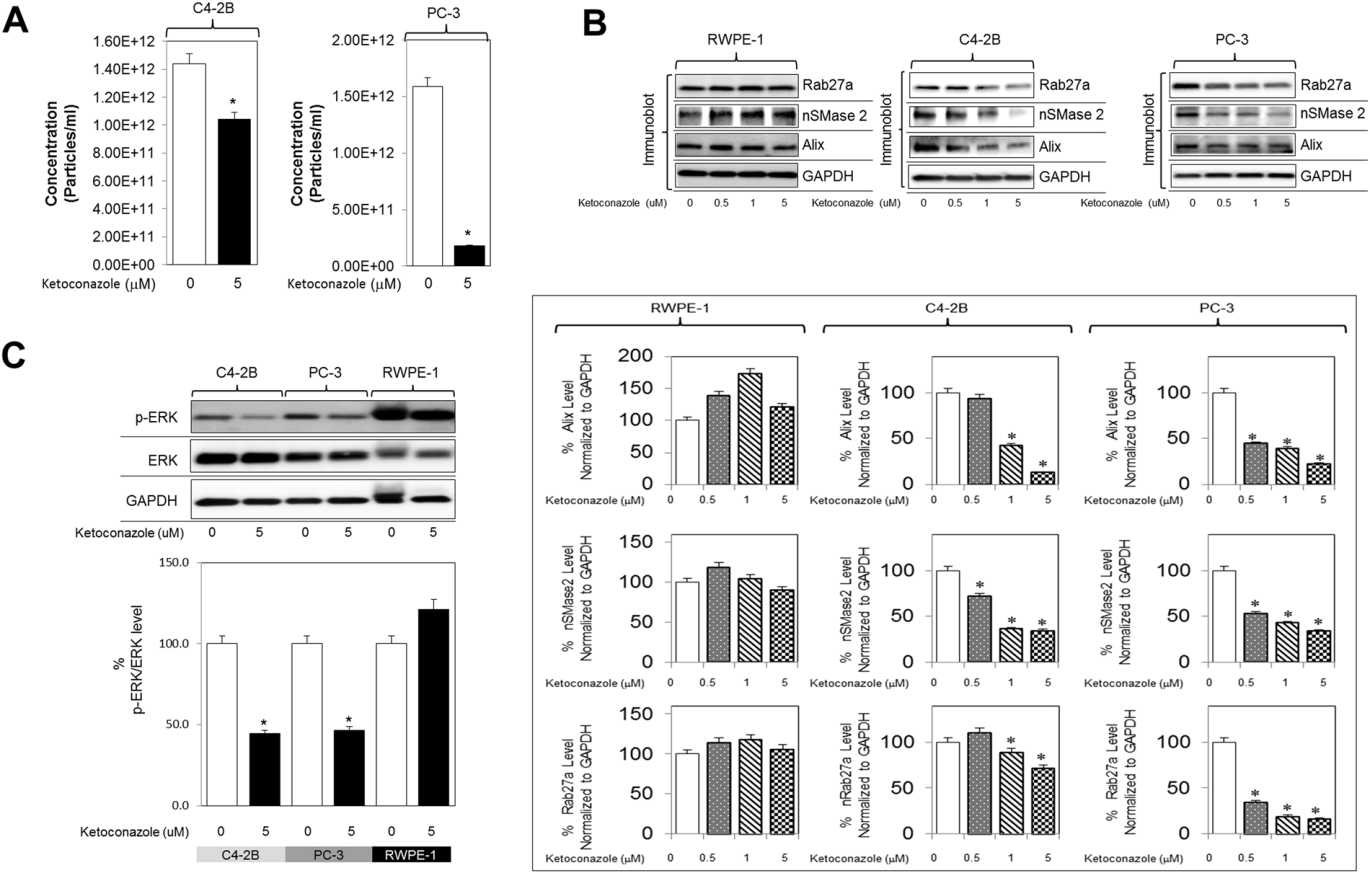
Ketoconazole (prototype imidazole) inhibits exosome biogenesis through ESCRT dependent and independent pathway. (**A**) C4-2B and PC-3 cells were maintained overnight in exosome free DMEM media and then treated with ketoconazole or DMSO (vehicle control) for 48 hours at 5 μM. Concentration of exosomes was measured by qNano IZON. Ketoconazole shows a significant decrease in the concentration of exosomes in C4-2B cells at 5 μM as well as in the PC-3 cells (**B**) Ketoconazole significantly inhibited the protein concentration of Alix, nSMase2 and Rab27a in a dose-dependent manner in C4-2B and PC-3 cells but not in the normal RWPE-1 cells. Full blots are presented in Supplementary Fig. S6. (**C**) Ketoconazole significantly inhibited activation p-ERK (downstream effector molecule of the Ras/Raf/ERK signaling pathway) but not total ERK in C4-2B, and PC-3 cells, but not in the RWPE-1 cells. Full blots are presented in Supplementary Fig. S6. Mean values and standard errors were derived from four independent experiments. *Denotes significance at p < 0.05 compared to controls and was calculated using GraphPad Prism.

Since neticonazole^32^ is not currently approved in the United States, we chose ketoconazole^33^ as another prototypical imidazole antifungal drug that is approved in the United States for PCa patients^34^. Ketoconazole (5 μM) decreased the concentration of exosome secreted by both C4-2B and PC-3 cells (Fig. 5A), whereas increasing concentrations (0-5 μM) elicited a robust dose-dependent decrease in Rab27a, nSMase2 and Alix in both C4-2B and PC-3 cells, but not in the normal RWPE-1 cells (Fig. 5B). Furthermore, studies evaluating the role of ERK signaling in the modulation exosome biogenesis and secretion, we observed that ketoconazole caused a significant decrease in p-ERK levels in both C4-2B and PC-3 cells, but not in RWPE-1 cells (Fig. 5C), suggesting it triggers its exosome-inhibitory effects via ESCRT-independent, ESCRT-dependent pathways, and Ras/ERK signaling pathways.

Table 2 summarizes the list of inhibitors validated as inhibitors in this study and includes manumycin A (from the recently published article on manumycin A^20^ from our group), tipifarnib, neticonazole, ketoconazole and climbazole.

**Table 2:** List of validated exosome inhibitors.

Compound (NCGC number, where applicable)	Structure	Parent MW (g/mol)	Therapeutic Information
MANUMYCIN A		550.652	• Farnesyl transferase inhibitor. (prevents the activation of Ras oncogenes, inhibits cell growth, induces apoptosis, and inhibits angiogenesis
TIPIFARNIB (NCGC00250406)		489.4	• Nonpeptidomimetic quinolinone with potential antineoplastic activity.
			• Farnesyl transferase inhibitor (prevents the activation of Ras oncogenes, inhibits cell growth, induces apoptosis, and inhibits angiogenesis).
			• R115777 is being studied in the treatment of AML and other types of cancer.
NETICONAZOLE (NCGC00249929)		302.436	• Antimycotic (antifungal)
			• Imidazole
KETOCONAZOLE		531.434	• Ketoconazole is an imidazole fungicidal agent with a very broad spectrum of activity against many fungal species that is used for treatment of superficial and systemic fungal infections
			• For the treatment of the following systemic fungal infections: candidiasis, chronic mucocutaneous candidiasis, oral thrush, candiduria, blastomycosis, coccidioidomycosis, histoplasmosis, chromomycosis, and paracoccidioidomycosis.
			• In clinical trials for treatment of Non hematologic malignancies, lymphoma and advamced cancers
CLIMBAZOLE (NCGC00166153)		292.763	• Antifungal
			• Inhibition of cytochrome P450
			• Antidandruff

Shown are drug name, NCGC number, structure, molecular weight and the available information on drug indication and mechanism of action reported in the PubChem (database of chemical molecules maintained by the National Center for Biotechnology Information).

## Discussion

Extracellular vesicles (EVs) (exosomes and MVs) are constantly released by both normal and cancerous cells^2,24^ and may be involved in cell-to-cell communications under both physiologic and pathologic conditions. However, exosomes are released at much higher levels than MVs^3^, and their biogenesis/secretion and ‘cargo’ contents are more regulated during cancer progression^4–7^. Exosomes modulate a multitude of signaling pathways in recipient cells through trafficking of cytosolic components and membrane proteins of the originating cells^24,35^ in various disease processes^36–38^, including progression of various types of cancers^35^. Cancer progression occurs due to efficient information exchange between the tumor cells and their stromal microenvironment^39^. Exosomes can both induce and facilitate a pro-tumoral microenvironment for the initiation of tumorigenesis^4,35^ and regulate the immune response to prime tumor progression and survival via promotion of angiogenesis, metastasis and drug resistance^1–4^. Our recent study demonstrated that tumor-secreted exosomes might also be critical in the neoplastic reprogramming of tumor-recruited stem cells^8^. However, despite significant advances in understanding the mechanisms linked to exosome biogenesis/release, there are currently no pharmacological agents to modulate exosome production from aggressive cancer cells. Therefore, the targeting of exosome-mediating physiological and pathological communications between cells will have significant therapeutic potential in a diverse array of diseases, including cancer.

New drug development and their regulatory approval take 10 to 20 years and it is a very expensive endeavor^40^. Drug repositioning, expanding a drug’s use to diseases other than that for which it was originally intended, is an alternative strategy to reduce both time and expense for drug “re-discovery” and advances in therapy^18^. In this regard, both the NPC^39,41,42^ and LOPAC^42–45^ libraries have been the cornerstone of recent drug repurposing discoveries, especially since they allow for flexible adaptation into high throughput assay for target validation. Our qHTS of >4,000 compounds identified 22 compound hits from the NPC and LOPAC collection that either suppress or activate exosome biogenesis and release by PCa cells. These clustered classes included, (a) antibiotics that cause cellular stress (two compounds); (b) Azole group of antifungals (four compounds); (c) β-adrenoreceptor agonists (seven compounds); and (d) several other agents that possess anti-inflammatory properties and antagonistic effects on specific receptors (eight compounds). Our validation assays with three different exosome detection/quantitation technologies and molecular analysis of key exosome regulatory pathways further established the efficacy of five potent lead candidates as exosome inhibitors, including two FTIs (manumycin A^20^ (MA) and tipifarnib), and three imidazoles (neticonazole, ketoconazole, and climbazole). Furthermore, our lead compounds have been previously tested in several pre-clinical models and, therefore, their potent ability to regulate exosome production by PCa cells may have a significant translational impact in drug-repositioning and clinical application as novel anticancer agents.

Previous studies have documented that MA has tumoricidal activity against different types of cancers, especially those with constitutively active Ras^21–23,46^. We recently reported that MA suppresses exosome biogenesis and secretion via targeted inhibition of Ras/Raf/ERK1/2 signaling in an hnRNP H1-dependent manner^20^. However, despite its efficacy, MA has not progressed into clinical trials due to its significant side effects. Our novel finding implicated the potential utility of the lead FTI compound, tipifarnib. Mutations in Ras/ERK pathway play pivotal roles in regulating multiple signaling pathways that are implicated in PCa growth, transformation, differentiation and stress response^47^. Current evidence supports the antitumor effects of tipifarnib, both *in vitro* and *in vivo*, and the safety and efficacy of this agent have been tested in several clinical trials for acute and chronic myeloid leukemia, breast cancer, glioblastomas, and melanomas^20–23,48^. Thus, in addition to its efficacy in inhibiting farnesylation, tipifarnib may exert its potent anti-tumor effects by suppressing exosome biogenesis and secretion by cancer cells.

Azole antifungals are a group of fungistatic agents with broad-spectrum activity, classified into two groups: the triazoles and the imidazoles. They all inhibit the cytochrome P450 dependent enzyme lanosterol 14-alpha-demethylase, which coverts lanosterol to ergosterol, the main sterol in fungal cell membrane^49,50^. However, some cross-inhibition of mammalian CYP51 by these azoles may also result in the reduction of cholesterol synthesis^51^, which may be a possible mechanism via which imidazoles may suppress exosome production. Our qHTS, for the first time, identified azole antifungals as potent suppressors of exosome biogenesis/release in the PCa cells. This includes three potent inhibitors (neticonazole, ketoconazole, and climbazole), which have been used for their antifungal activities due to their suppressive effects on ergosterol biosynthesis and activator (nitrefazole) of exosome biogenesis and secretion. Most interestingly, nitrefazole’s therapeutic effects are due to a different mechanism than the other four azoles. Imidazole antifungals are potent inhibitors of 14-alpha-demethylase, a CYP450 Phase-I drug-metabolizing enzyme that is highly enriched within the mitochondria^32,33^. On the other hand, nitrefazole is a potent inhibitor of aldehyde dehydrogenase (ALDH), which increases the side-effects profile of alcohol, and is thus used in the treatment of alcoholism. An important point to note is that ALDH is a Phase-II drug metabolizing enzyme present in the cytosol. Azoles may also regulate cholesterol-metabolism, by binding to another P450 enzyme, CYP46A1^52^. Thus, one direct inhibitory effect of azoles in PCa may be due to their ability to inhibit cholesterol biosynthesis. However, it is also noteworthy that MVs, and not exosomes, are formed at plasma membrane lipid-rafts that are enriched in cholesterol^25,53^. Therefore, the cholesterol-inhibitory effects of imidazoles may not only be directly associated with their suppressive function on exosome biogenesis and secretion, but also in MV production of MVs. Whether azoles modulate exosome production by regulating either Phase-I (CYP51) or Phase-II (ALDH) enzymes certainly warrant further investigation.

The four antifungal azoles identified by qHTS have shown significant side-effects and drug-drug interaction, and thus, have not been clinically approved. Therefore, we tested the efficacy of a clinically approved imidazole antifungal drug, ketoconazole, that has been shown to have a strong affinity towards human CYP51^51,52^. We showed that similar to both neticonazole and climbazole, potent inhibition of exosome biogenesis was possible using low-dose ketoconazole. Furthermore, we observed that ketoconazole inhibited Alix, nSMase2, and Rab27a, which would, in turn, lead to suppression of multiple pathways towards exosome biogenesis and secretion. Thus, azoles having lesser side effects but possessing high exosome inhibitory effects may be repositioned as anti-cancer agents.

The activators of exosome biogenesis discovered in our study (sitafloxacin, forskolin, SB218795, fenoterol, nitrefazole and pentetrazol) were validated by at least two independent methods. However, their underlying mechanisms of action remain to be elucidated. Exosome biogenesis activators may have significant therapeutic implications in conditions such as immunoregulation. For example, EVs derived from immunosuppressive dendritic cells (DCs) generated *in vitro* have been shown to reverse early onset of collagen-induced arthritis. Also, APC- and MSC-derived exosomes play an important immunosuppressive role in transplantation. In addition, in a clinical trial for malignant glioma, tumor exosome-loaded DCs were shown to stimulate a tumor-specific CD8+ CTL response against autologous tumor cells showing some evidence of cancer specific immunostimulation^54^.

Overall, we believe that the discovery of clinically approved drugs, such as the farnesylation inhibitor tipifarnib, and the well-studied antifungal drug ketoconazole as potent inhibitors of exosome biogenesis and/or secretion from aggressive PCa cells will enable their rapid progression as adjunct therapy in the clinic. Our findings address a significant un-met need, as there are currently no available drugs that target the continuous production of deleterious exosomes in cancer patients. The long-term goal will be to test the *in vivo* efficacy of inhibitors and activators of exosome biogenesis/release in cancer cells as a synergistic approach to curb cell-to-cell communication and disease progression in multiple types of advanced cancers. Last, but not the least, our lead compounds are also expected to be beneficial for exosome modulation under physiologic conditions and other disease conditions.

## Materials and Methods

### Materials

RPMI-1640 medium, penicillin/streptomycin solution and fetal bovine serum (FBS) were purchased from Invitrogen (Camarillo, CA, USA). The NPC drug library was custom assembled at National Center for Advancing Translational Sciences (NCATS, NIH, Bethesda, MD, USA). The LOPAC collection was purchased from Sigma (Sigma-Aldrich, St. Louis, MO, USA). Unless otherwise indicated, all other drugs were purchased from commercial sources.

### Cell culture and plasmids

The metastatic castration-resistant C4-2B cells were a kind gift from Dr. Leland W. Chung (Cedar-Sinai Medical Center, Los Angeles, CA, USA). The human prostate cancer epithelial metastatic cell line PC-3 (ATCC CRL-1435) and the normal prostate epithelial cell line RWPE-1 (ATCC CRL-11609) were purchased from ATCC (Manassas, VA, USA). All cell lines were authenticated by short tandem repeat (STR) profiling (Genetica DNA Laboratories, Burlington, NC, USA). The cells were cultured in RPMI-1640 medium supplemented with 10% fetal bovine serum, 2 mM L-glutamine and 1% penicillin/streptomycin (P/S) and RWPE-1 cells were maintained in K-SFM media with supplements. For routine maintenance, each cell line was grown as a monolayer at 37° with 5% carbon dioxide and 95% relative humidity (RH). For collection of conditioned media (CM), the cells were trypsinized, plated and allowed to attach overnight in complete media, following which the media were changed to FBS-free media for 48 hours and the supernatants were collected for exosome isolation. For treatment with the compounds, the cells were trypsinized, plated and allowed to attach overnight in complete media, following which the cells were treated with various concentrations of the compounds in exosome-free FBS supplemented media and then harvested at the indicated times for subsequent analysis. Cell viability was measured by the MTT (methylthiazolyldiphenyl-tetrazolium bromide) (Sigma-Aldrich) cell cytotoxicity assay according to the manufacturer’s protocol as we described^55^. The plasmid (CD63-GFP) (Origene cat# RG201733) was used to generate the stable C4-2B-CD63-GFP cells.

### CD63-GFP Biogenesis Assay

Exosome biogenesis was measured using the intracellular GFP signal from CD63-GFP-expressing C4-2B cells. A 5 µL cell suspension of 2000 cells/well (in RPMI containing 10% serum and 1% Geneticin) were added to wells in 1536-well poly-D-lysine coated plates with a Multidrop™ Combi Reagent Dispenser (Thermo Fisher Scientific Inc., Waltham, MA, USA) and incubated for 48 hours at 37 °C in 5% CO_2_ with 95% RH. Media was then removed from the wells, replaced with 5 µL serum-free Opti-MEM (Thermo Fisher Scientific Inc.) and 1% Geneticin and returned to the incubator for overnight incubation at 37 °C in 5% CO_2_ with 95% RH. A Pin-Tool based compound dispenser (Kalypsys Inc., San Diego, CA, USA) was used to add 23 nL of compound-solutions in DMSO, and the plates were incubated for 96 hours at 37 °C in 5% CO_2_ with 95% RH. Fluorescence from GFP expressing cells was quantitated with an Acumen laser scanning imaging cytometer (TTP Labtech Inc., Cambridge MA, USA). The Acumen software was used to measure the total number and intensity of fluorescent objects.

### Quantitative High Throughput Screen (qHTS) and hit selection

For the screen, the measured GFP signal intensity for each well was normalized to the median fluorescence intensity per cell from the DMSO control wells as 100% signal and fluorescence intensity per cell from control wells with compound at an EC_100_ as 0% signal. The activity of the hits from the qHTS screen was determined based on the Curve Response Class (CRC) classification from dose response qHTS, in which normalized data is fitted to a 4-parameter dose-response curve using a custom grid-based algorithm to generate CRC score for each compound dose- response^56^. CRC values of ±1.1, ±1.2, ±2.1, ±2.2 are considered highest quality hits; CRC values of ±1.3, ±1.4, ±2.3, ±2.4 and ±3 are weak and inconclusive hits; and a CRC value of 4 are inactive compounds. The miniaturization and optimization of a qHTS compatible assay are shown in Supplementary Fig. S2C and D.

### Exosome isolation

Exosomes from the CM of C4-2B cells treated with the compounds were isolated by a modified differential ultracentrifugation protocol, as we previously reported^8^ and as shown in Supplementary Fig. S3. Briefly, the CM was centrifuged at 300 × g for 10 min to remove contaminating cells and then centrifuged at 20,000 × g for 30 min to remove larger microvesicles (MVs). The resulting supernatants were then transferred to fresh tubes and filtered through 0.8 μm filter GVS Maine Poretics, PCTE Filter Membranes (Thermo Scientific, Pittsburgh, PA, USA). The filtered samples were centrifuged for 2 hours at 110,000 × g to pellet the enriched exosomes (Beckman Coulter, Brea, CA, USA). Pellets were then re-suspended in PBS and centrifuged at 100,000 × g for another 1 hour. The exosome pellets were then re-suspended in 150~300 μL of PBS and stored in aliquots at −80 °C until used.

### Analysis of EV particles by the qNano IZON system

As we described previously^20^, we employed the TRPS technology (qNano IZON system; Izon, Cambridge, MA, USA) to measure the concentrations, size-distribution and diameters of the extracellular vesicles (EVs; exosomes and MVs) in the CM of C4-2B-CD63 GFP cells treated with the 22 lead compounds or control vehicle (DMSO). The system was calibrated for voltage, stretch, pressure, and baseline current using two standard beads: CPC100B (mode diameter: 114 nm, concentration: 1.0E13/ml) and CPC70D (mode diameter: 70 nm, concentration: 3.0E13/ml). A diluted sample size of 40 μL and NP100 nanopore (for 50–200 nm size range) were used and data analysis was performed by a qNano IZON software.

### Analysis of exosomes by flow cytometery

Exosomes derived from CM of C4-2B- CD63-GFP cells were maintained in exosome-free FBS media and treated with the compounds or DMSO was analyzed by flowcytometer. The MACSQuant Analyzer 10 system (Miltenyi Biotech Inc., San Diego, CA, USA) is configured with Forward (FSC) and Side Scatter (SSC) channels off the 488 nm laser and includes fluorescent channels with detection channels based of excitations from 405 nm, 488 nm, and 638 nm lasers. To optimize the detection of EV’s, an SSC trigger was set to reduce electronic noise with 0.22 μm Millex-HV Syringe Filter (EMD Millipore, Billerica, MA, USA) and using the filtered non-EV containing control samples (PBS). A fluorescent trigger was also used on the B1 channel which has 488 nm excitation and a bandpass filter allowing 525/50 nm wavelengths of emitted light. This allowed further reduction of electronic noise. Nano Fluorescent standard particles (Cat. No. NFPPS-52-4K, Spherotech Inc. Lake Forest, IL, USA) were used as a reference standard to calibrate the experiment based on particle size and fluorescence. The MACSQuant Analyzer 10 syringe driven fluidics system allows for the volumetric measurement of samples that can be quantified on an events/µL or events/mL basis. In addition, a NanoFACS system consisting of ZE5 Cell Analyzer (BioRad) was also used to analyze the GFP signal of CD63-GFP-expressing C4-2B cells. The NanoFACS is configured with up to five spatially separated lasers including the 355, 375, 405, 488, 561 and 640 nm lasers. With this configuration, a 561-nm SSC threshold was set to allow a stable rate of background scattered light detection from the fluidics stream at the instrument noise floor. The 488 nm SSC (Side SCatter) and FSC (Forward SCatter) were used to detect scattered light from the exosomes and reference beads (with a resolution range of ~80–500 nm, as defined by control particles). The GFP signal of the secreted CD63-GFP particles representing the exosomes was also measured at 523 nm. The X-axis measures the FSC-GFP signal, whereas the Y-axis measures the SSC parameter.

### Immunoblot analyses

Protein extracts were subjected to immunoblot analysis using antibodies against Alix, p-ERK, ERK (Cell Signaling Technology, Danvers, MA, USA), GAPDH (Santa Cruz Biotechnology, Dallas, TX), Rab27a (Proteintech, Chicago, IL, USA) and nSMase2 (Santa Cruz Biotechnology). Immune complexes were detected with appropriate secondary antibodies from Santa Cruz Biotechnology and chemiluminescence reagents (Pierce, Rockford, IL, USA) as we described^54^. Immunoblot signals were captured using the Image Quant Las 300 (GE Healthcare, Piscataway, NJ, USA). Densitometric analysis was performed using ImageJ (NIH, Bethesda, MD, USA, http://imagej.nih.gov/ij/).

### Immunofluorescence

Cells were seeded on glass chamber slides and treated as described in the figure legends. Cells cultured on glass coverslips were washed twice with PBS (pH 7.2) and fixed under permeabilized conditions (4% PFA) for 30 min at room temperature. After fixation, cells were washed in PBS and incubated at room temperature with normal goat serum to block nonspecific binding. The coverslips were incubated overnight at 4 °C with primary mouse anti-CD63 (Abcam, Cambridge, MA, USA), rinsed with PBS and incubated with the appropriate fluorescently-labeled secondary antibodies; mouse Ab Texas red (Red) and then examined under a Nikon fluorescence microscope, as previously described^57^.

### Statistical analysis

Data are presented as means ± S.E.M. of three or more independent experiments performed in triplicate. For western blots, a case representative experiment is depicted in the figures section. Statistical analyses were performed using GraphPad Prism5 (GraphPad Software Inc., La Jolla, CA, USA). Either the two-sided *t*-test or analysis of variance (ANOVA) was performed. *P*-values less than 0.05 were considered significant.

## Electronic supplementary material

Supplementary Information
