# Supplementary material for: High-throughput screening identified selective inhibitors of exosome biogenesis and secretion: A drug repurposing strategy for advanced cancer
Source: Sci Rep. 2018 May 25;8:8161. doi: 10.1038/s41598-018-26411-7 (PMC5970137; doi:10.1038/s41598-018-26411-7)
Supplement: Supplementary file 1 — Supplementary Information [file 41598_2018_26411_MOESM1_ESM.pdf]

High-throughput screening identified selective inhibitors of exosome biogenesis and secretion: A drug repurposing strategy for advanced cancer

Amrita Datta*[,1], Hogyoung Kim*[,1], Lauren McGee*[,4], Adedoyin Johnson[1], Sudha Talwar[1], Juan Marugan[4], Noel Southall[4], Xin Hu[4], Madhu Lal[4], Debasis Mondal[2,3], Marc Ferrer[4], Asim B. Abdel-Mageed[1,2,3]

Departments of Urology[1] and Pharmacology[2] and Tulane Cancer Center[3], Tulane University School of Medicine, New Orleans, LA 70112.

[4] Division of Preclinical Innovation, National Center for Advancing Translational Sciences (NCATS), National Institutes of Health, Bethesda, Maryland 20850, United States.

Supplementary Information

(Figures and Tables)

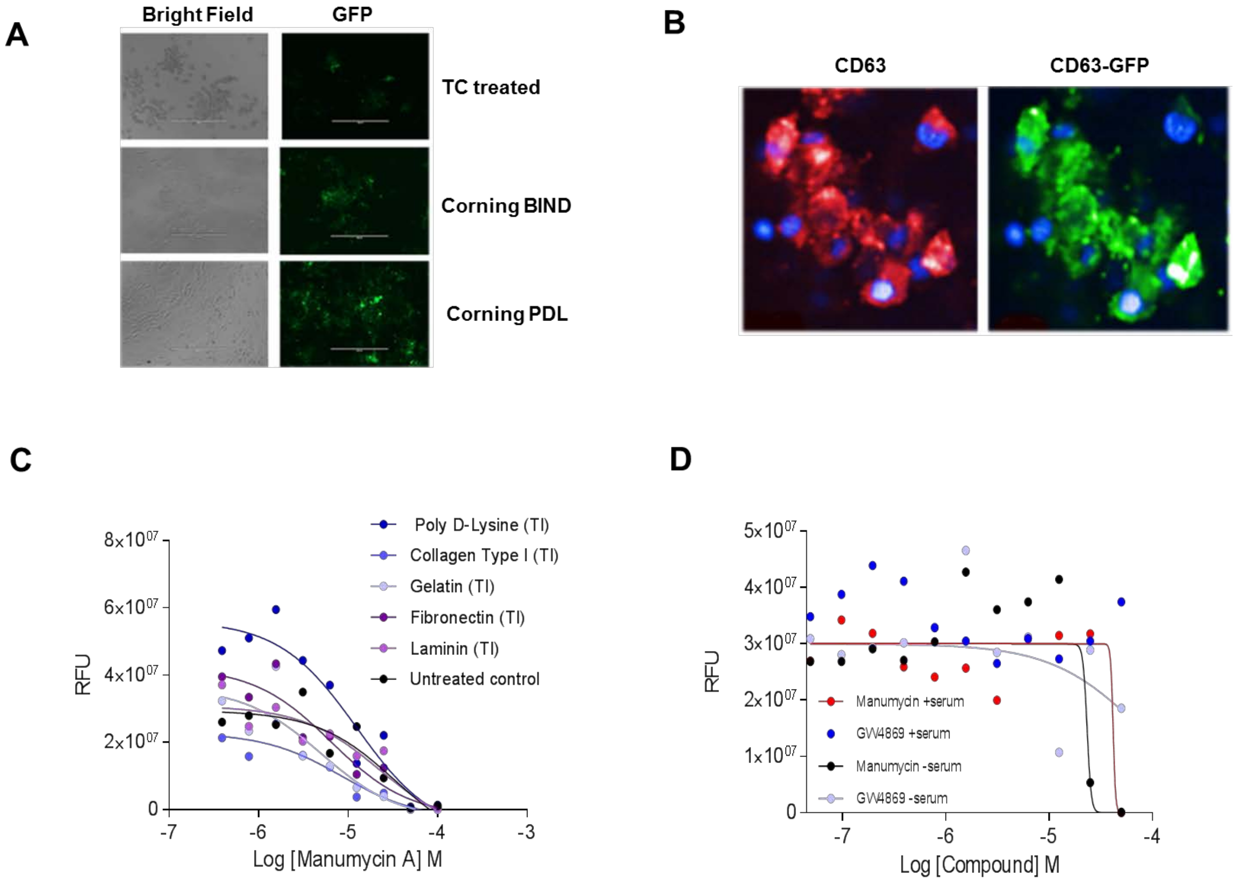

Supplemental Figure S1. Optimization of plates for screening in a response qHTS format. (**A**) C4-2B-CD63-GFP expressing cells were plated on a different plate for 24 hours. The images were captured GFP images using Leica fluorescence microscope. (**B**) Optimized assay conditions included the seeding, coating of the plates with poly-D-lysine-coated, seeding 2,000 cells/well for 96 hours. The left panel was subjected to immunofluorescence staining with antibodies against CD63. Right panel was image of CD63-GFP expressing cells. The images were captured using Leica fluorescence microscope. (**C**) C4-2B-CD63-GFP cells were seeded on the plates for 24 hours in plates pre-coated with different coating solutions, such as poly-D-lysine, collagen TypeI, gelatin, fibronectin, and laminin. The cells were treated with various concentrations of manumycin A (MA) or control vehicle (DMSO) for 48 hours. The CD63-GFP

RFU signals were analyzed by qHTS system. (**D**) C4-2B-CD63-GFP cells were growing on the PDL-coated plates with or without serum for 24 hours. The cells were treated with a various concentrations of MA (a farnesyltransferase inhibitor), and GW4869 (a neutral sphingomyelinase inhibitor) or control vehicle (DMSO) for 48 hours. The CD63-GFP RFU signals were analyzed by the qHTS system.

Figure S2

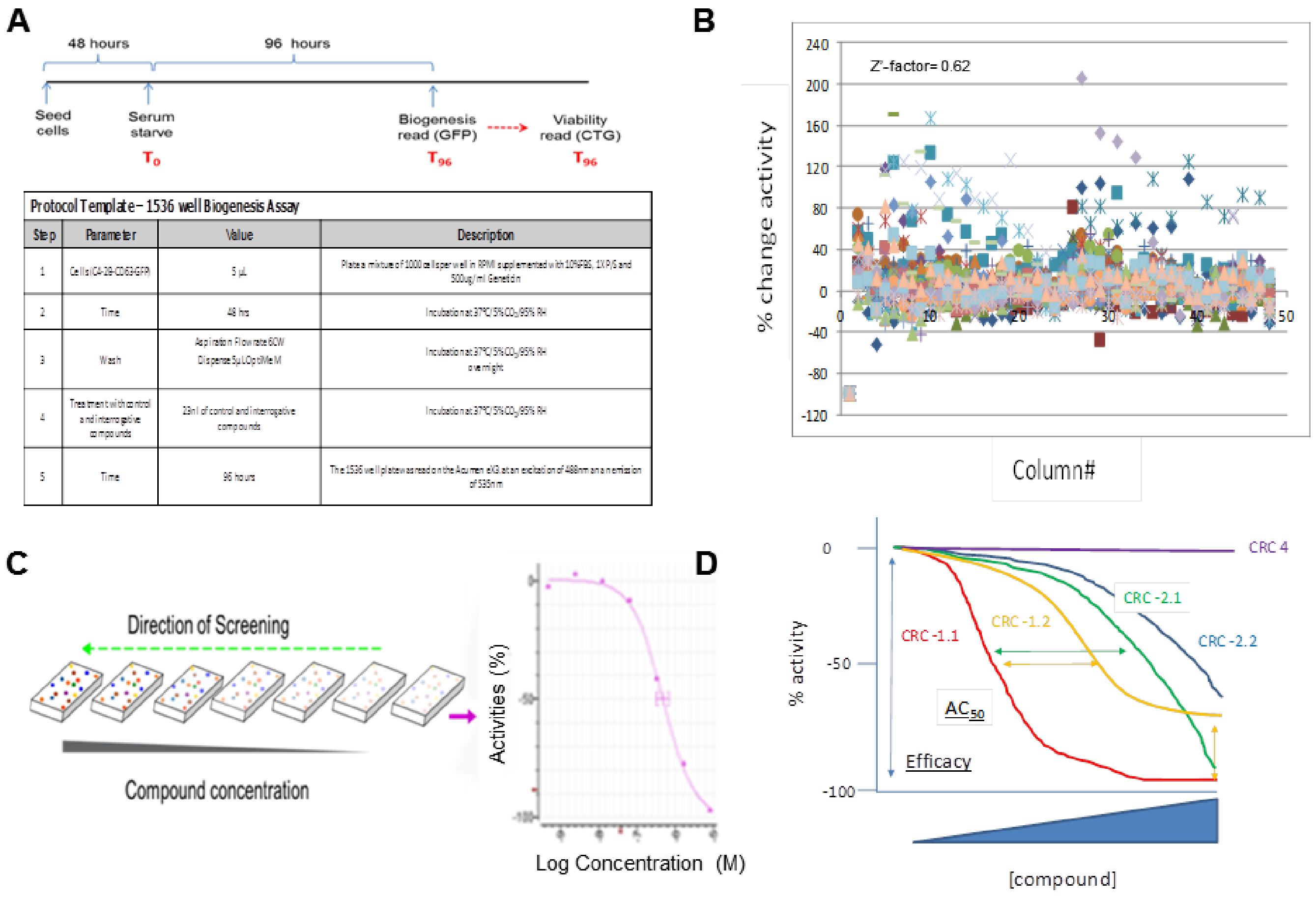

Protocol Template – 1536 well Biogenesis Assay			
Step	Parameter	Value	Description
1	Cells (C4-2B-CD63-GFP)	5 µL	Plate a mixture of 1000 cells per well in RPMI supplemented with 10%FBS, 1X P/S and 500ug/ml Geneticin
2	Time	48 hrs	Incubation at 37°C/ 5% CO_2/95% RH
3	Wash	Aspiration Flowrate 60W Dispense 5µL OptiMeM	Incubation at 37°C/ 5% CO_2/95% RH overnight
4	Treatment with control and interrogative compounds	23nl of control and interrogative compounds	Incubation at 37°C/ 5% CO_2/95% RH
5	Time	96 hours	The 1536 well plate was read on the Acumen eX3 at an excitation of 488nm an a n emission of 535nm

Supplemental Figure S2. Developing a high throughput assay for detection of exosome biogenesis. (**A**) Time Line: C4-2B-CD63-GFP cells were added to 1536 well plates, serum starved after 48 hours, compounds were added 24 hours later, and biogenesis/viability were read 96 hours later. Miniaturization and Optimization of a qHTS compatible assay (**B**) Miniaturization and multiplexing various assay readouts to get the most informative informational output. The high content imaging assay was run on the Acumen eX3, a high content laser scanning cytometer, at both 24 hours and 96 hours post serum starvation to read both GFP intensity and the number of nuclei. (**C**) Optimization of compounds for screening in a dose response qHTS format. 1536-well CD63-GFP based assay was developed to measure exosome concentrations using the intracellular GFP signal from CD63-GFP expressing C4-2B cells. The GFP signal intensity measured for each well was normalized to the median

fluorescence intensity per cell from the DMSO control wells as 100% signal and fluorescence intensity per cell from control wells with compound at an EC_{100} as 0% signal. This CD63-GFP based prescreening of the NPC and LOPAC libraries was used to identify the Inhibitor/Activator status of drugs and probable IC50 of GFP activity. Detailed description of the protocols, software and instruments used are described in “Materials and Methods” section. (**D**) Schematic depicting examples of dose response curves and curve response class (CRC) scores.

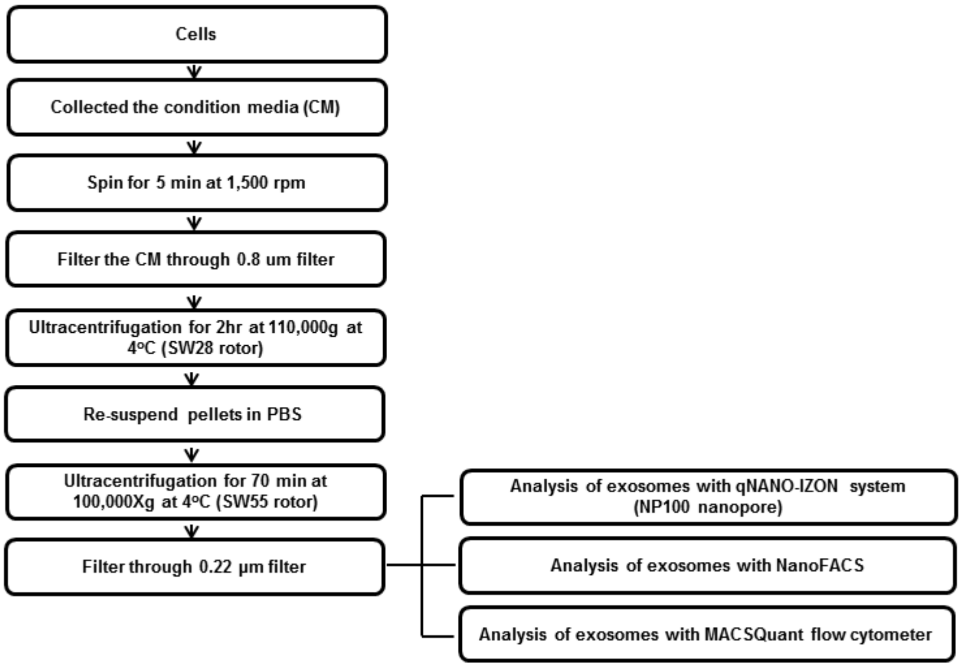

Supplemental Figure S3. Flowchart of isolation and analysis of the extracellular vesicles (EVs). Purification and analysis of EVs including exosomes and microvesicle (MVs), in the conditioned media (CM) of C4-2B-CD63-GFP cells treated with DMSO (vehicle) or all of drugs were performed according to the depicted flow chart. Following differential ultracentrifugation, EVs were prepared by filtration through 0.22 μm filters, respectively, and analyzed by qNano IZON^{TM} system using NP100 (size range: 50-200 nm) nanopores, respectively.

Figure S4

Sr. no	Name	Percentage of Total Events	Count	Count/ml	Total Count/ml = (Count/ml X Dilution Factor)
1	PBS	100.00	76	5.21e3	5.21e3
	PBS-GFP	2.63	2	137	137
2	NO-FL-CONTROL* [C4-2B exosomes (no CD63-GFP)]	100.00	499	3.34e4	3.34e4
	NO-FL-CONTROL-GFP	2.61	13	869	869
3	CONTROL [C4-2B-CD63-GFP exosomes)	100.00	114486	7.61e6	7.61e6
	CONTROL-GFP	62.64	71717	4.77e6	4.77e6
4	Sitafloxacin (diluted 1:10)	100.00	79244	5.27e6 (1:10)	5.27e7
	Sitafloxacin-GFP (diluted 1:10)	71.58	56720	3.77e6 (1:10)	3.77e7
5	Forskolin	100.00	212026	1.41e7	1.41e7
	Forskolin-GFP	58.16	123324	8.2e6	8.2e6
6	Ketoprofen	100.00	89942	5.98e6	5.98e6
	Ketoprofen-GFP	58.05	52209	3.47e6	3.47e6

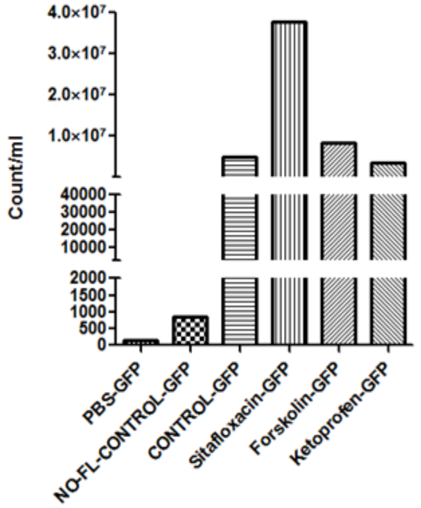

Supplemental Figure S4. MACSQuant™ data: C4-2B-CD63-GFP cells cultured in exosome free RPMI media were treated with activators of exosomes identified by the qNano IZON™ in Figure 1 (sitafloxacin, forskolin, and ketoprofen) for 48 hours. The exosomes in the conditioned media were isolated and analyzed by the MACSQuant™ Analyzer 10 Flow Cytometer as described in the "Materials and Methods" section. Treatments were at concentrations of 10 μM for all other compounds. All the compounds identified as activators using the qNano IZON™ were validated as potent activators of exosome biogenesis by the MACSQuant™ as well (with the exception of Ketoprofen). Three controls were used in this experiment. PBS without EVs was used as a reference control. The NO-FL-CONTROL are exosomes from parental C4-2B cells that don't express CD63-GFP cells and is used as no-fluorescence control. Exosomes from DMSO treated C4-2B-CD63-GFP is used as GFP control. The percentage of total events, count and count/ml of the samples are tabulated.

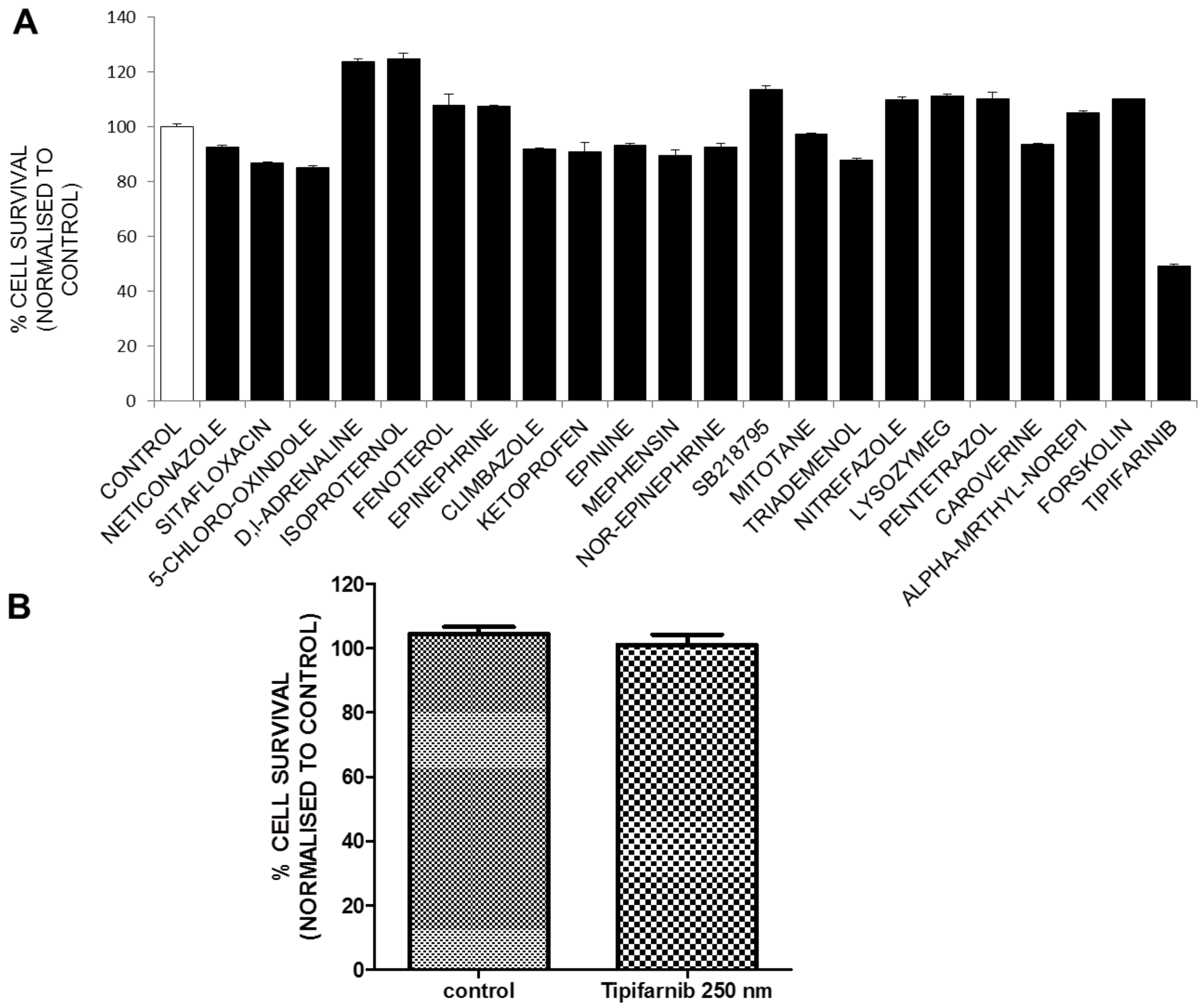

Supplemental Figure S5. Cell viability assays. (**A**) After 72 hours drug exposure with 10 μM concentrations, except for tipifarnib (1 μM), cells were incubated with 10 μl of 5 mg/ml of MTT reagent for 4 hours. Absorbance values were measured at 570 nm. (**B**) A representative viability assay on C4-2B cells treated with Tipifarnib at 250 nM concentration for 48 hours. Viability was assessed with MTT assay in triplicate for each cell type, with appropriate controls. Data represent normalized mean values.

For Figure 3B

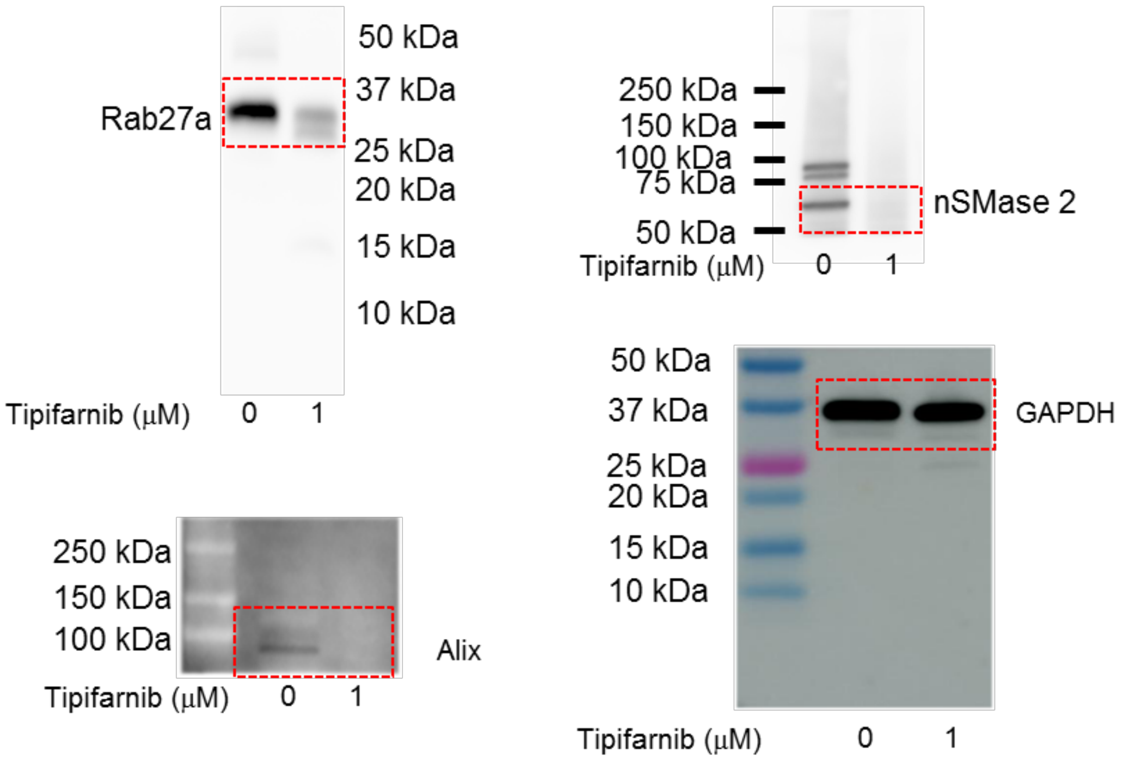

Supplemental Figure S6. Full-length gels and blots used for Figure 1-5.

For Figure 3C

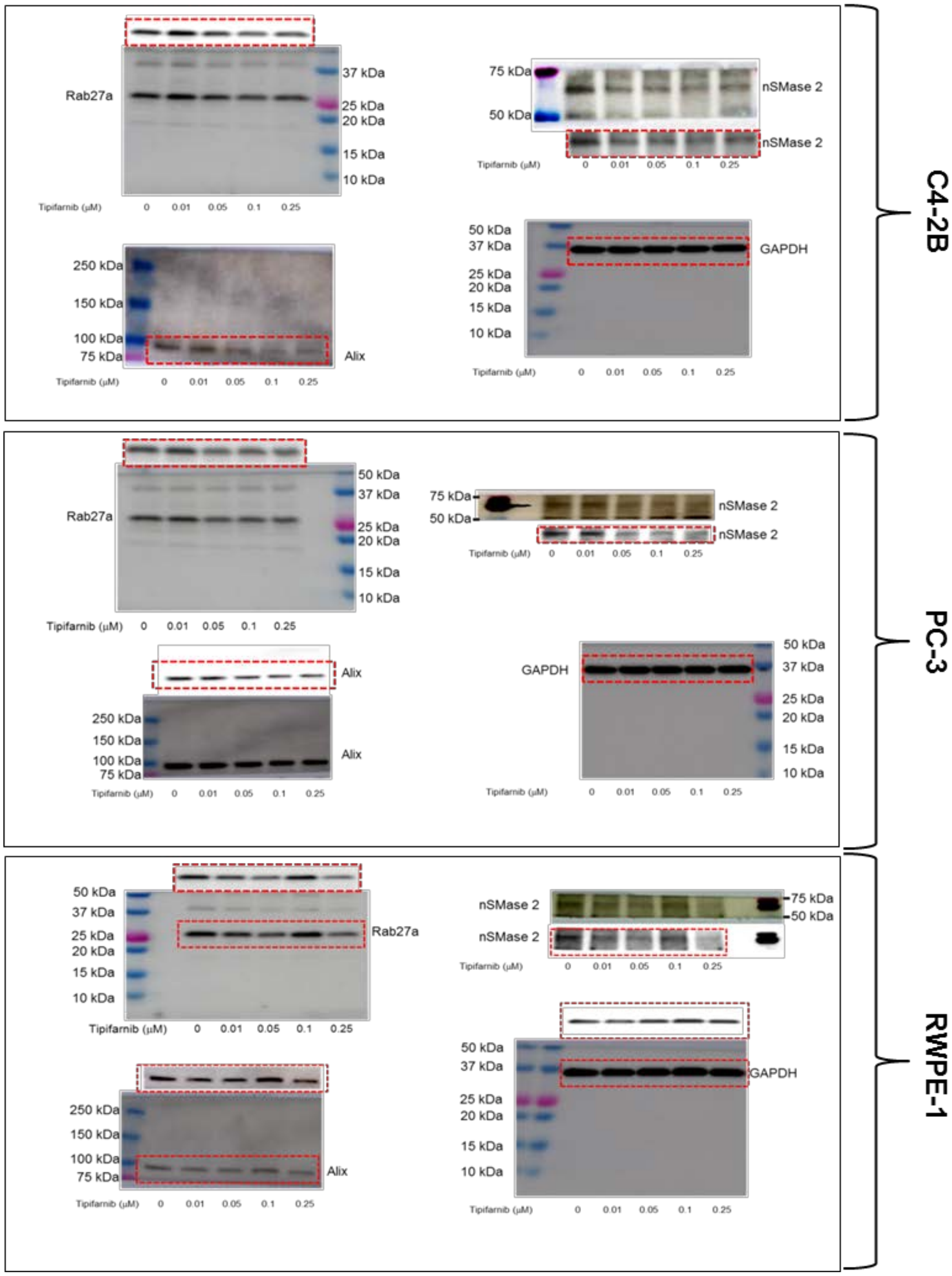

Supplemental Figure S6. Full-length gels and blots used for Figure 1-5.

For Figure 3D

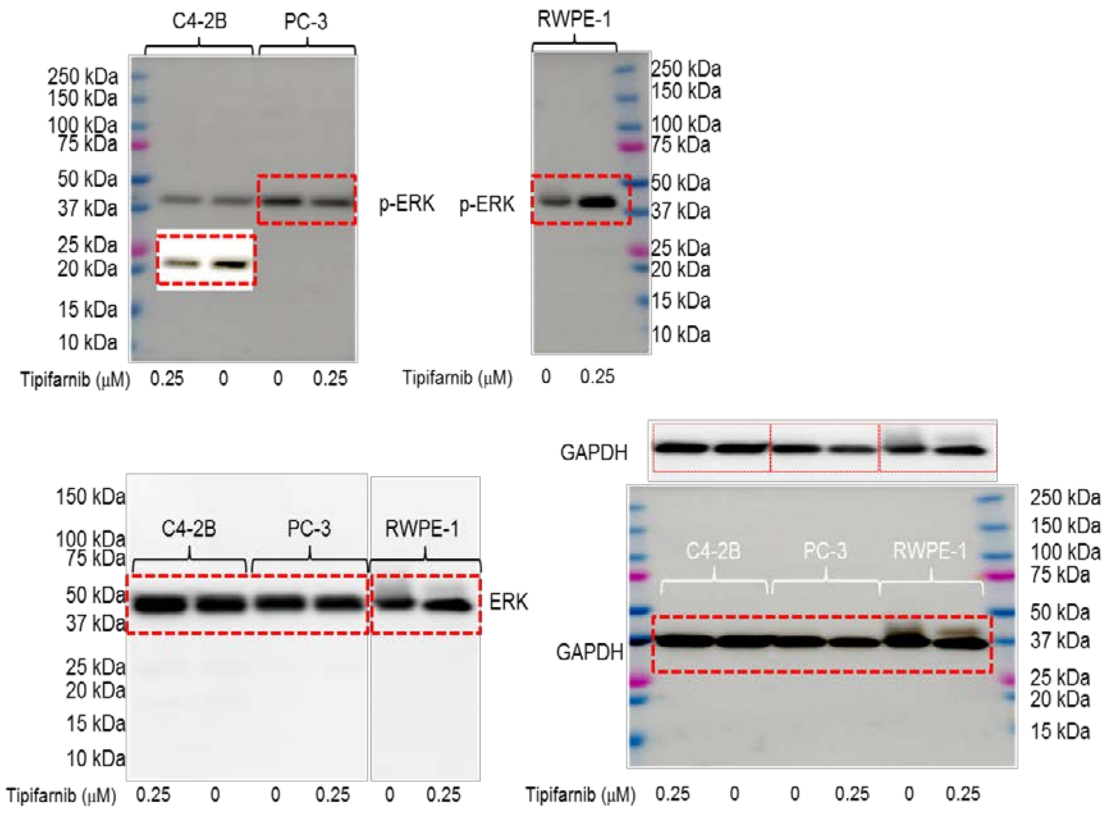

Supplemental Figure S6. Full-length gels and blots used for Figure 1-5.

For Figure 4B

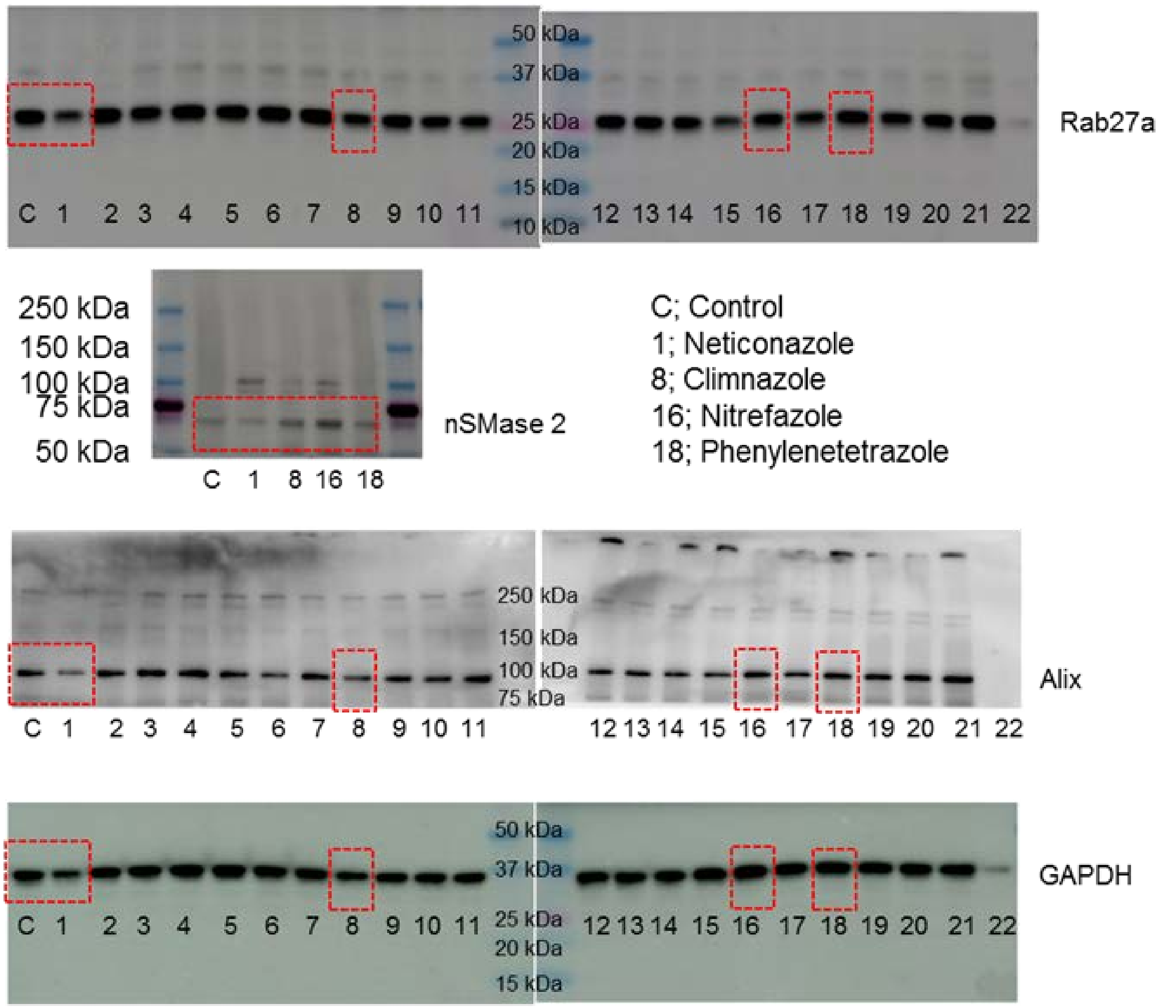

Supplemental Figure S6. Full-length gels and blots used for Figure 1-5.

For Figure 4C

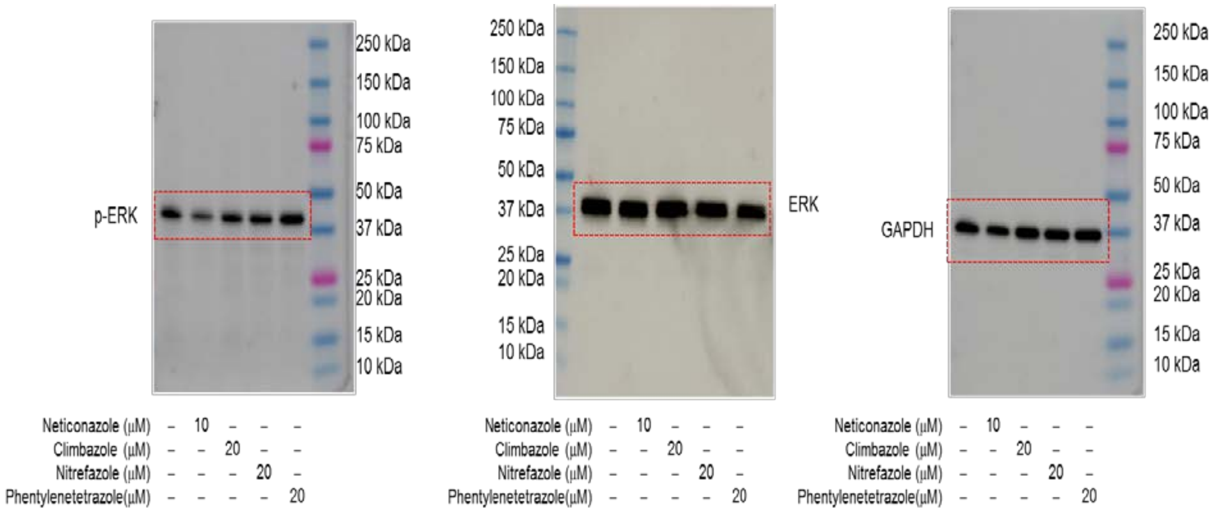

Supplemental Figure S6. Full-length gels and blots used for Figure 1-5.

For Figure 4E

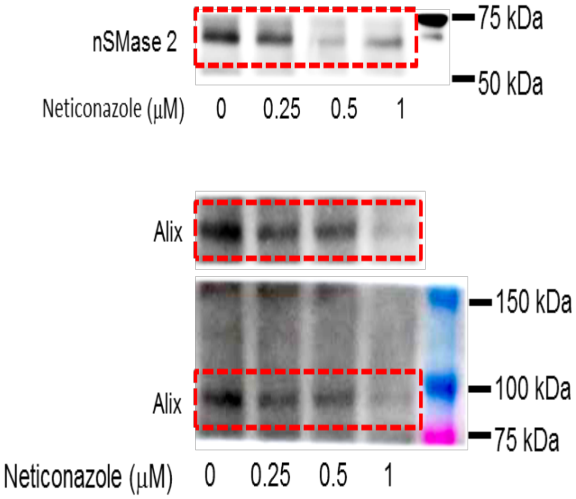

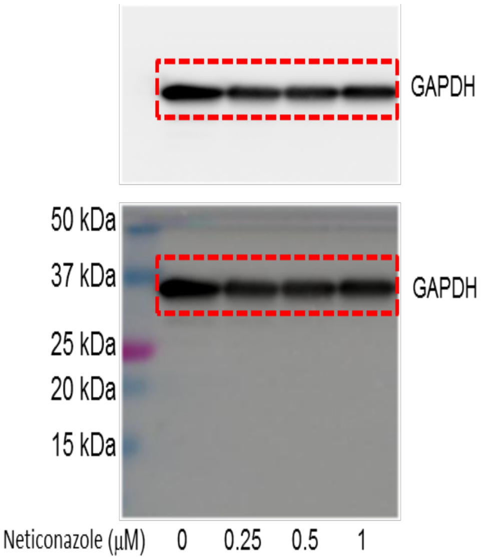

Supplemental Figure S6. Full-length gels and blots used for Figure 1-5.

For Figure 5B

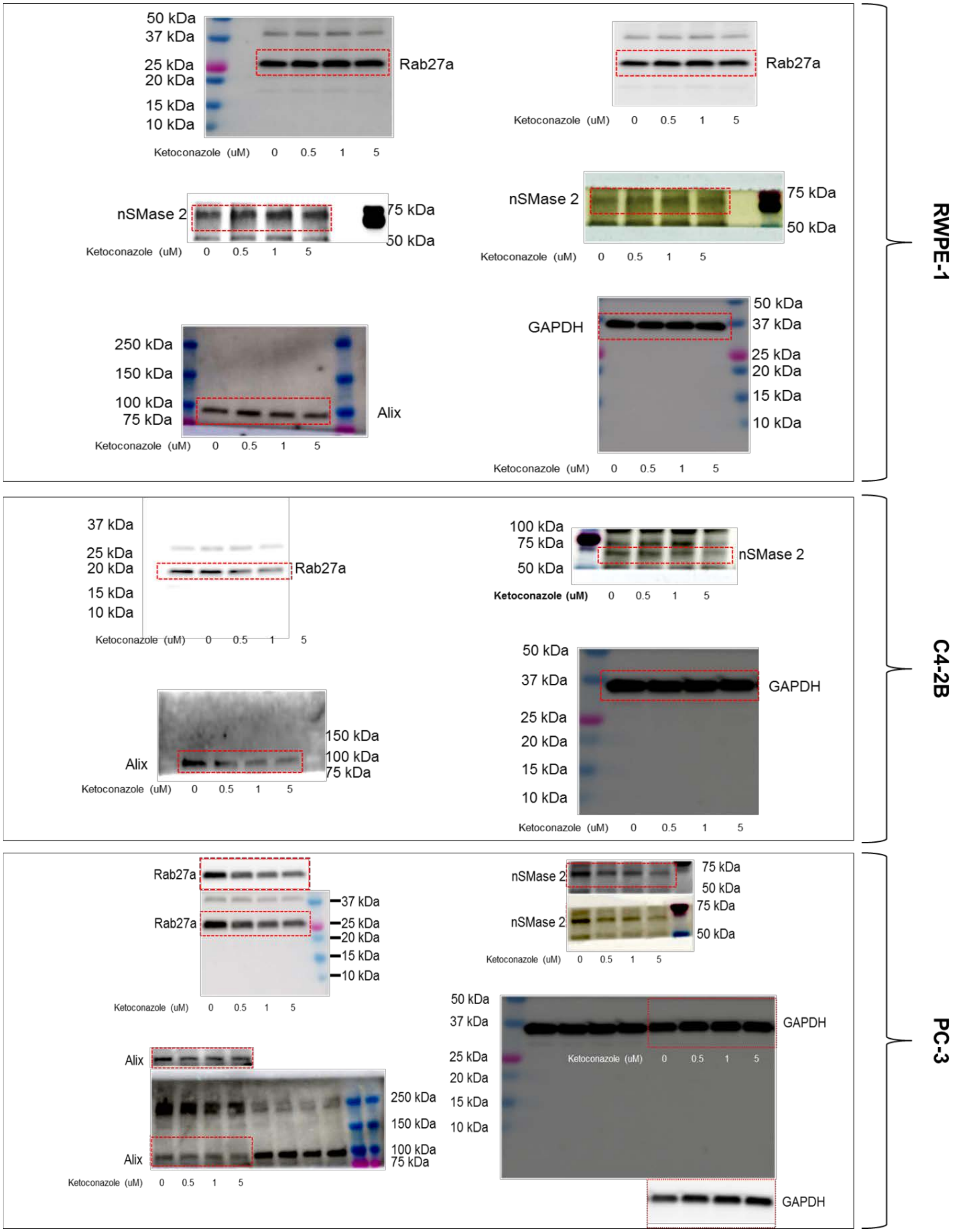

Supplemental Figure S6. Full-length gels and blots used for Figure 1-5.

For Figure 5C

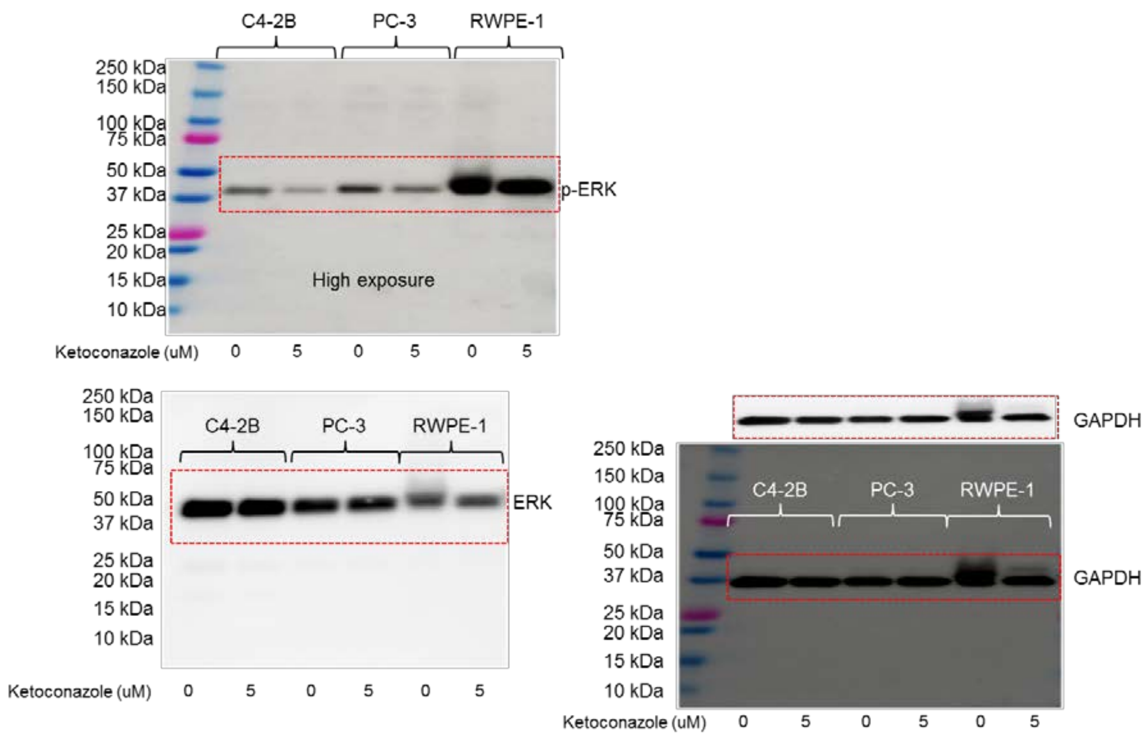

Supplemental Figure S6. Full-length gels and blots used for Figure 1-5.

	NCGC #	Name	Particle Diameter Mean (nm)	STDEV	Particle Diameter Mode (nm)
0		Control	115	42.3	81
1	NCGC00249929-01	Neticonazole	123	53.6	92
2	NCGC00183844-04	Sitafloxacin	120	49.4	79
3	NCGC00253720-01	Chloroxindole	110	35.2	89
4	NCGC00015417-04	Racepinephrine	116	45.1	84
5	NCGC00179436-03	Isoproterenol	111	44.1	85
6	NCGC00015430-06	Fenoterol Hydrobromide	121	51	93
7	NCGC00142615-05	L-epinephrine bitartate	114	44.9	88
8	NCGC00166153-03	Climbazole	106	32.9	85
9	NCGC00015578-14	Ketoprofen	128	62.2	88
10	NCGC00015355-04	N-Methyldopamine	124	47.5	102
11	NCGC00094908-05	Mephensin	122	51.5	80
12	NCGC00159406-12	Norepinephrin Bitartate	110	43.6	78
13	NCGC00025131-04	SB 218795	111	42.7	85
14	NCGC00015226-13	Mitotane	128	50.5	89
15	NCGC00066947-05	Triadimenol	120	58.1	83
16	NCGC00183023-01	Nitrefazole	110	42	76
17	NCGC00181761-01	Lysozyme	111	39.6	92
18	NCGC00015827-11	Pentylenetetrazole	120	48.4	81
19	NCGC00186046-01	Caroverine	113	40.8	80
20	NCGC00093795-05	Methylnorepinephrine	111	39.6	81
21	NCGC00024996-31	Forskolin	104	32.4	85
22	NCGC00250406-04	Tipifarnib	120	49.4	79

Supplemental Table S1. Effect of drugs on particle diameter of C4-2B CD63-GFP cell-secreted EVs. qNano IZON^{TM} analysis depicting the diameter and diameter mode of exosomes secreted by C4-2B CD63-GFP cells transfected with plasmid.
